# Asgard archaea shed light on the evolutionary origins of the eukaryotic ubiquitin-ESCRT machinery

**DOI:** 10.1038/s41467-022-30656-2

**Published:** 2022-06-13

**Authors:** Tomoyuki Hatano, Saravanan Palani, Dimitra Papatziamou, Ralf Salzer, Diorge P. Souza, Daniel Tamarit, Mehul Makwana, Antonia Potter, Alexandra Haig, Wenjue Xu, David Townsend, David Rochester, Dom Bellini, Hamdi M. A. Hussain, Thijs J. G. Ettema, Jan Löwe, Buzz Baum, Nicholas P. Robinson, Mohan Balasubramanian

**Affiliations:** 1 Centre for Mechanochemical Cell Biology, Division of Biomedical Sciences, Warwick Medical School, University of Warwick, Coventry, CV4 7AL UK; 2 Division of Biomedical and Life Sciences, Faculty of Health and Medicine, Lancaster University, Lancaster, LA1 4YG UK; 3 MRC Laboratory of Molecular Biology, Cambridge, CB2 0QH UK; 4 Laboratory of Microbiology, Wageningen University, 6708 WE Wageningen, The Netherlands; 5 Department of Aquatic Sciences and Assessment, Swedish University of Agricultural Sciences, SE-75007 Uppsala, Sweden; 6 Department of Chemistry, Lancaster University, Lancaster, LA1 4YB UK; 7 Present Address: Department of Biochemistry, Indian Institute of Science, Bangalore, India

**Keywords:** X-ray crystallography, Membrane proteins, Archaeal evolution, ESCRT

## Abstract

The ESCRT machinery, comprising of multiple proteins and subcomplexes, is crucial for membrane remodelling in eukaryotic cells, in processes that include ubiquitin-mediated multivesicular body formation, membrane repair, cytokinetic abscission, and virus exit from host cells. This ESCRT system appears to have simpler, ancient origins, since many archaeal species possess homologues of ESCRT-III and Vps4, the components that execute the final membrane scission reaction, where they have been shown to play roles in cytokinesis, extracellular vesicle formation and viral egress. Remarkably, metagenome assemblies of Asgard archaea, the closest known living relatives of eukaryotes, were recently shown to encode homologues of the entire cascade involved in ubiquitin-mediated membrane remodelling, including ubiquitin itself, components of the ESCRT-I and ESCRT-II subcomplexes, and ESCRT-III and Vps4. Here, we explore the phylogeny, structure, and biochemistry of Asgard homologues of the ESCRT machinery and the associated ubiquitylation system. We provide evidence for the ESCRT-I and ESCRT-II subcomplexes being involved in ubiquitin-directed recruitment of ESCRT-III, as it is in eukaryotes. Taken together, our analyses suggest a pre-eukaryotic origin for the ubiquitin-coupled ESCRT system and a likely path of ESCRT evolution via a series of gene duplication and diversification events.

## Abstract (web summary)

The ESCRT pathway is crucial for membrane remodelling in eukaryotes. Here, Hatano et al. explore the phylogeny, structure, and biochemistry of homologues of the ESCRT machinery and the associated ubiquitylation system in Asgard archaea, the closest living relatives of eukaryotes.

## Introduction

The ESCRT (Endosomal Sorting Complex Required for Transport) machinery is composed of several protein complexes and associated accessory proteins, including the ESCRT-0, -I, -II, -III subcomplexes, Vps4, and ALIX/Bro1^1–18^ (Supplementary Fig. 1). These proteins act in sequence to bind, deform and cut membranes during membrane trafficking^2–5^, cell division^19,20^, viral egress^21,22^ and in other important topologically similar membrane remodelling events in eukaryotes^23–39^. When driving the formation of multivesicular bodies (MVBs), where the role of ESCRT in trafficking has been best characterized, the ESCRT machinery is recruited to endosomal membranes by ubiquitylated transmembrane proteins that are targeted to vesicles^40^. The ubiquitin (Ub) moiety is recognized by ESCRT-0 and -I subcomplexes^9,14,41,42^. ESCRT-I proteins together with the ESCRT-II subcomplex then corral these ubiquitylated transmembrane proteins into membrane domains^9,11^. Finally, the ESCRT-II subcomplex nucleates the local formation of ESCRT-III co-polymers which, through action of the Vps4 AAA-ATPase, undergo structural changes to drive membrane invagination and scission to release vesicles containing the Ub-marked transmembrane proteins^43–53^.

ESCRT-I, -II, -III components are found conserved across the eukaryotic lineages, pointing to this machinery being present in the last eukaryotic common ancestor (LECA), while ESCRT-0 is only encountered in Opisthokonts^54^. Homologues of ESCRT-III and Vps4 are coded by the genomes of many archaeal species^55–60^, and in some systems function in archaeal membrane remodelling during cytokinesis and virus release^61^. More recently, PspA and Vipp1 have been recognized as bacterial ESCRT-III related proteins^62–64^. These observations suggest that a subset of ESCRT components have more ancient evolutionary origins. Finally, the identification of a full complement of ubiquitin and its activating enzymes (E1, E2, and E3) in some archaeal species has provided evidence for ubiquitylation cascades functioning in protein degradation in the archaeal ancestors of eukaryotes^58,65,66^.

This begs the question: when in evolution did ESCRT-I and ESCRT-II machineries arise, and when was ubiquitylation co-opted to regulate ESCRT? Metagenome assemblies of the recently discovered Asgard archaea, the closest living relatives of eukaryotes, have revealed that homologues of the entire ubiquitylation cascade, ESCRT-III (and Vps4), and components of the ESCRT-I and ESCRT-II subcomplexes are all encoded by the genomes of these archaea^57,58^. However, validating this conclusion in cells is currently very difficult, as only one Asgard member has been isolated and cultured^67^; and its growth rate, physiology and the lack of essential tools currently prevent its use as a cell biological model.

To circumvent these challenges, here we apply a diverse set of experimental approaches to characterise Asgard archaeal homologues of the eukaryotic Ub-ESCRT system, focusing on the ESCRT-I and ESCRT-II subcomplexes. Our analysis shows that, like its eukaryotic counterparts, the Asgard ESCRT-I subcomplex stably recognises ubiquitin. Furthermore, by carrying out a comprehensive two-hybrid analysis, we have been able to identify protein-protein interactions within and between the different ESCRT subcomplexes. Additionally, our data show that Asgard ESCRT subcomplexes have likely arisen through a process of gene duplication and diversification, prior to the evolution of more complex eukaryotic ESCRT assemblies. Taken together, this work reveals the presence of a multi-component ubiquitin-associated ESCRT pathway that predates the emergence of the eukaryotic ESCRT machinery.

## Results

### Phylogenetic analyses indicate that Asgard archaeal genomes encode homologues of most, but not all components of the Ub-ESCRT machinery

**Fig. 1:**
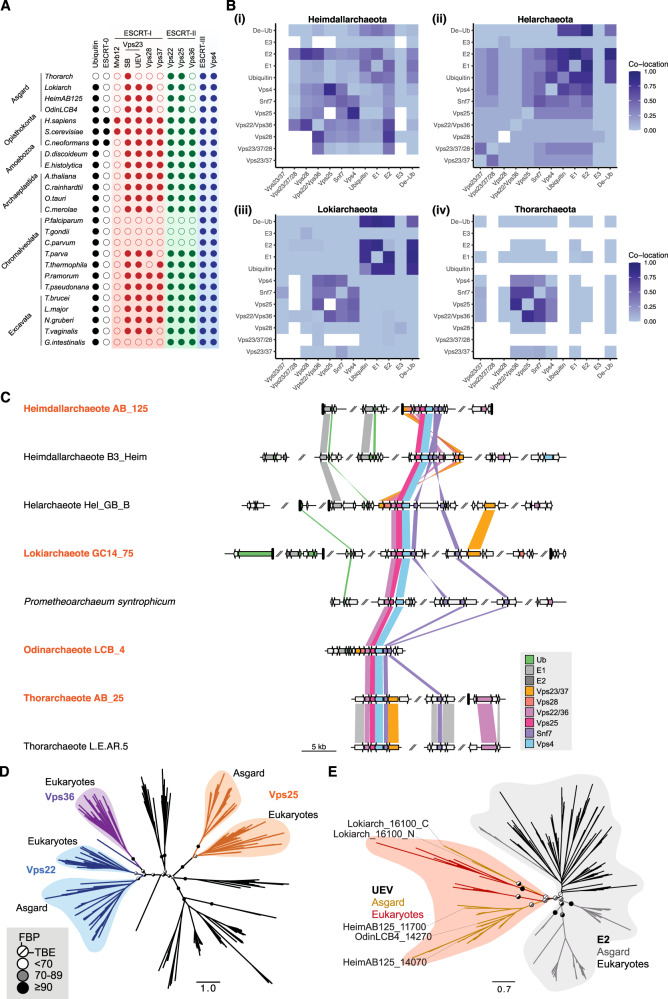
The genomes of Asgard archaea, Lokiarchaeota, Heimdallarchaeota, Helarchaeota, and Odinachaeota possess homologues of the ubiquitin-ESCRT pathway. **A** List of proteins in the Asgard archaea and eukaryotic Ubiquitin-ESCRT (Ub-ESCRT) pathway. **B** Co-location of Ub/ESCRT protein-encoding genes in Heimdall- (i; 22 genomes), Hel- (ii; 9 genomes), Loki- (iii; 29 genomes) and Thorarchaeota (iv; 30 genomes). A colour gradient indicates the fraction of genomes in which a pair of genes was found to co-locate within a region of <10 kb. White cells indicate gene pairs not found co-existing in any Asgard genome analysed. **C** Synteny plot of selected genomes. Arrows represent genes and are coloured if their products are annotated as containing diagnostic domains for Ub/ESCRT proteins (see Methods). Homologues of Vps23/37 (as determined via alignment (Supplementary Fig. 6)) in the vicinity of the ESCRT gene cluster in Helarchaeote Hel_GB_B, Heimdallarchaeote B3_Heim and *Ca*. Odinarchaeum yellowstonii LCB_4 only possess E2/UEV domains. A gene encoding a fusion of Vps23/37 and Vps28 is coloured as both orange and red. Genome regions are plotted up to a distance of 2 kb from ubiquitin or ESCRT protein-encoding genes (coloured), or until a contig boundary (thicker vertical lines). Similarity lines indicate best-reciprocal BLAST-p^134^ hits with an e-value lower than 1e-5. The names of the organisms used for experimental analyses in later sections are marked in orange. **D** Phylogenetic reconstruction of Vps22 and Vps36. Unrooted maximum likelihood phylogenetic tree of Vps22 (blue), Vps36 (purple) and Vps25 (orange) and outgroup (black) sequences. The tree was reconstructed using IQ-Tree^103^ under the LG + C60 + R4 + F + PMSF model. Support values are only shown for the deeper branches connecting gene homologues and represent standard Felsenstein bootstrap proportions (upper left) or Transfer-Bootstrap Expectation (TBE) (lower right) values based on 100 bootstrap pseudoreplicates. The full tree with all leaf and support labels is shown in Supplementary Fig. 4. **E** Phylogenetic reconstruction of UEV domain-containing proteins and E2 ubiquitin-conjugating enzymes. Unrooted maximum likelihood phylogenetic tree of the UEV domain-containing proteins (gold [Asgard], red [Eukarya]) and E2 ubiquitin-conjugating proteins (grey [Asgard], black [Eukarya]) in Eukarya and Asgard archaea, respectively. The tree was reconstructed using IQ-Tree under the Q.pfam+C20 + G4 + F + PMSF model. Support values are only shown for the deeper branches, following the same pattern as in **D**.

Many of the recently discovered Asgard archaeal genomes encode a wide array of so-called ‘eukaryotic signature proteins’ (ESPs) and appear unique amongst prokaryotes in possessing close homologues of most of the proteins that make up the ESCRT-I and -II complexes, together with ESCRT-III and Vps4 and homologues of ubiquitin and the associated ubiquitin-modification enzymes^57,58^. While these data suggest the possibility of some Asgard archaea possessing functional Ub-ESCRT membrane trafficking machinery, it is noticeable that Asgard archaeal genomes appear to lack a number of genes encoding proteins essential for ESCRT function in eukaryotes (Fig. 1A). Thus, to further explore how these ESCRT components might operate in Asgard archaeal systems, we began by generating a catalogue of proteins with homology to the components of the eukaryotic Ub-ESCRT pathway in distinct Asgard phyla (Fig. 1A, Supplementary Fig. 2, and Supplementary Fig. 3).

We focused on diverse Asgard archaeal species identified from published metagenomic assemblies^57,58^. Within such genomes, as previously described^57,58^, we were able to identify genes coding for close homologues of ubiquitin, ubiquitin modifying enzymes, ESCRT-I components, ESCRT-II subunits, together with homologues of ESCRT-III and Vps4 (Fig. 1A and Supplementary Fig. 3)^58,68^. These analyses strongly suggest that many Asgard archaea possess a bona fide eukaryote-like ESCRT system.

Since gene clustering in prokaryotes frequently brings together genes with common functions, we sought to determine the extent to which ubiquitylation and ESCRT-related genes are found co-located within specific regions of Asgard archaeal genomes (Fig. 1B, C, and Supplementary Fig. 4). As previously described^58^, within a single Odinarchaeota genome (now referred to as *Ca*. Odinarchaeum yellowstonii LCB_4, following the recent closure of the Odinarchaeota LCB_4 metagenomic assembly into a single chromosome contig^69^) the full set of putative gene-products with homology to *Ub-ESCRT* were found together within a single gene cluster (Fig. 1C). We extended this analysis by developing a simple metric of gene clustering, which we then applied to Heimdall-, Loki-, Thor-, and the more recently described Helarchaeotal genomes, all of which harbour genes encoding ESCRT proteins^57,58,68^. This was achieved by measuring the fraction of genomes within each phylum in which each pair of genes co-localises within less than 10 kb (Fig. 1B; white indicates no evidence of co-location and deep purple indicates full co-location; further details of these analyses are described in the Methods section). This analysis revealed that the entire set of genes was clustered together in Hel- and Heimdallarchaea genomes, and was organized into two relatively discrete Ub and ESCRT genomic regions in Lokiarchaeota (Fig. 1B). In addition, we observed a consistent pattern of association across genomes in which the genes for ESCRT-III and Vps4 were found most tightly associated with homologues of Vps25 (Fig. 1B, C, and Supplementary Fig. 4). This is striking as Vps25 is the subunit of the ESCRT-II complex in eukaryotes that recruits ESCRT-III to membranes, triggering vesicle budding^1–18^. Vps22/Vps36 homologues (ESCRT-II components) were found to have a similar but slightly less consistent pattern of co-location with ESCRT-III and Vps4, and were usually found closely associated with Vps25 (Fig. 1B, C, and Supplementary Fig. 4).

During this analysis, we also noted that, whereas Vps22 and Vps25 function in eukaryotes as part of a single hetero-tetrameric complex together with a divergent Vps22 homologue, Vps36, Asgard archaeal genomes possess a single gene encoding a Vps22/Vps36-like protein (Fig. 1A and C, and Supplementary Fig. 3). A phylogenetic analysis was used to confirm that the Asgard Vps22-like protein is a closer homologue of Vps22 than it is of Vps36 (Fig. 1D, Supplementary Fig. 5) and to confirm the presence of a distinct Vps25 protein in both Asgard archaea and eukaryotes.

Asgard archaeal genomes also code for clear homologues of the eukaryotic ESCRT-I machinery, including homologues of Vps23 (which contain a Ubiquitin E2 variant or “UEV” domain) and Vps28, both of which possess a Steadiness Box domain (SB) (Supplementary Fig. 3). Interestingly, these genes were not tightly clustered in Asgard archaeal genomes (Fig. 1B, C). Furthermore, the organization of this set of genes was variable across lineages, including genomes in which individual domains were brought together to form fusion proteins (Fig. 1B, C, Supplementary Figs. 6–8). In addition, Asgard archaea were found to lack a clear homologue of the eukaryotic Vps37. This ESCRT-I subunit is a homologue of Vps23 (Supplementary Figs. 6–8)^9,70–72^, implying that Vps37 may have arisen later in evolution in the branch leading to eukaryotes. In summary, with the notable exception of the Thorarchaeota, which seemingly lack true ubiquitin homologues (Fig. 1A–C and Supplementary Fig. 3), most Asgard archaeal genomes have the potential to encode proteins that together resembles large parts of the conserved eukaryotic Ub-ESCRT system.

### Asgard archaeal ESCRT-I subcomplexes bind ubiquitin

**Fig. 2:**
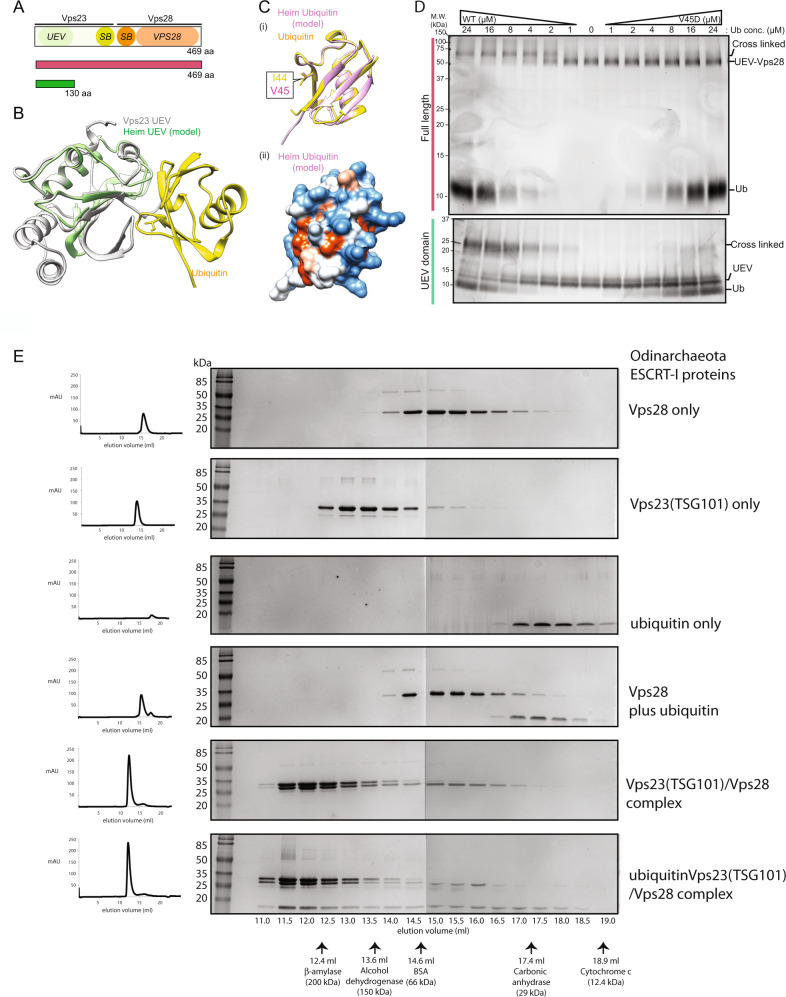
Assembly of Asgard ESCRT-I complexes with ubiquitin binding to the UEV domain of Vps23(TSG101). **A** Schematic diagram of the domain structure of HeimAB125 Vps28 and the truncation design used in the experiments. The N-terminal ubiquitin E2 variant domain (“UEV”), the steadiness box (SB), and the core domain of Vps28 (“Vps28”) identified previously are highlighted. **B** A model of the three-dimensional structure of HeimAB125 UEV. The three-dimensional structure model was created using AlphaFold 2 by templating the structure of the budding yeast Vps23 bound to ubiquitin. Vps23 (PDB: 1UZX [https://www.rcsb.org/structure/1UZX]^113^, chain A, light green) and HeimAB125 UEV (model structure, purple) are superimposed. Ubiquitin bound to Vps23 (PDB: 1UZX [https://www.rcsb.org/structure/1UZX]^113^, chain B) is shown in yellow. **C** The structural model of HeimAB125 ubiquitin generated using AlphaFold 2 was superimposed with the structure of ubiquitin in complex with Vps23 (PDB: 1UZX [https://www.rcsb.org/structure/1UZX]^113^, chain B). Ubiquitin (PDB: 1UZX [https://www.rcsb.org/structure/1UZX]^113^, chain B, yellow), model structure of HeimAB125 ubiquitin (light blue), Vps23 (PDB: 1UZX [https://www.rcsb.org/structure/1UZX]^113^, chain A, light green) were shown. An amino acid residue (Val45) on the model structure located in the ubiquitin hydrophobic patch, which is important for ubiquitin-UEV interactions, is highlighted in magenta. The structure of HeimAB125 ubiquitin is illustrated in the ribbon diagram (i) and the surface model (ii). **D** HeimAB125 UEV binds ubiquitin dependent on a hydrophobic patch. The interaction between HeimAB125 ubiquitin (wild-type or V45D mutant) and UEV-Vps28 (full-length, top panel) or UEV domain (bottom panel) was tested by BS3-mediated chemical crosslinking, followed by SDS-PAGE to detect the increase in their molecular weight. Three experimental repeats performed with representative experiment displayed. Source data are provided as a Source Data file. **E** Size-exclusion chromatography analysis of the thermophilic Odinarchaeota ESCRT-I subcomplex assembly. All proteins and complexes were incubated at 60 °C for 10 min before analysis. From top to bottom: Vps28 protein only (top); Odin Vps23 protein only; ubiquitin only; Vps28 pre-incubated with ubiquitin (no interaction); Odin Vps23 pre-incubated with Vps28 (stable complex formation); Odin Vps23 pre-incubated with Vps28 and ubiquitin (bottom—ubiquitin binds to the Odin Vps23 / Vps28 complex, via the UEV domain of Odin Vps23. For additional controls see Supplementary Fig. 10). All proteins were separated on a Superdex S200 HR 10/300 size-exclusion chromatography column. The relative elution volumes of the size standards β-amylase (200 kDa), alcohol dehydrogenase (150 kDa), bovine serum albumin (BSA) (66 kDa) and carbonic anhydrase (29 kDa) and cytochrome-c (12.4 kDa) are also indicated in grey. Eluted fractions were resolved by SDS-PAGE and visualized by Coomassie stain. Left: chromatography UV traces (at 280 nm) for the respective elution profiles. Three experimental repeats performed with representative experiment displayed. Source data are provided as a Source Data file.

In eukaryotes, a variant of the ubiquitin E2 (UEV) domain plays a key role in Ub recognition by ESCRT-I^73^. The UEV domain is similar in structure to the E2 region of the ubiquitin-conjugating E2 enzymes, but lacks a key catalytic cysteine. We identified several similar proteins in Asgard archaea. The Heimdallarchaeota AB125 genome codes for four E2-like candidates: HeimAB125_07740, HeimAB125_09840, HeimAB125_11700 and HeimAB125_14070 (Supplementary Fig. 7A, B). Structural models of these Heimdallarchaeotal proteins were generated using AlphaFold 2^74,75^ (Supplementary Fig. 7A). Structural superimpositions were used to confirm that while two of the Heimdallarchaeotal proteins (HeimAB125_07740 and HeimAB125_09840) possess putative catalytic Cys residues (characteristic of bona fide E2 ubiquitin-conjugating proteins), two (HeimAB125_11700 and HeimAB125_14070) did not contain cysteine residues at the expected catalytic positions, raising the possibility that these may have UEV domains and function as ubiquitin-binding proteins (Supplementary Fig. 7A and B). One of these UEV domain-containing proteins represents the fusion of a Vps28 domain to a UEV-Vps23-like structure (containing ESCRT-I signatures: coiled-coil stalk region and a steadiness box). This Heimdallarchaeota protein may therefore harbour both Vps23-like ubiquitin-binding activity (via the UEV-domain) and Vps28-like functions, which are central to ESCRT-II subcomplex recruitment (Fig. 2A–C and Supplementary Figs. 7 and 8). A phylogenetic analysis was used to confirm that both Asgard and eukaryotic UEV-like proteins cluster with eukaryotic Vps23 homologues and away from bona fide ubiquitin E2 enzymes – suggesting a divergence in the structure and function of UEV and E2 domains that predates the Last Asgard and Eukaryotic Common Ancestor (Fig. 1E). Other Asgard species, most notably *Ca*. Odinarchaeum yellowstonii LCB_4, possess separable and distinct Vps23-like (containing the UEV domain) and Vps28 proteins, as seen in eukaryotes (Supplementary Fig. 8). Both these Odinarchaeotal proteins contain steadiness boxes, which in eukaryotes are critical for the assembly of the ESCRT-I subcomplexes^9,72,76^.

To test for physical interactions between these putative ESCRT-I proteins, we recombinantly expressed in *E. coli* and purified Heimdallarchaeota and Odinarchaeota ubiquitin together with their corresponding putative UEV-containing proteins and performed in vitro binding experiments. In the case of the Heimdallarchaeota proteins, the interaction between the purified full-length UEV-Vps23-Vps28 fusion protein and the corresponding Heimdallarchaeota ubiquitin homologue was analysed by chemical crosslinking followed by SDS-PAGE (Fig. 2D). This revealed an increase in the apparent molecular weight of the protein upon ubiquitin binding and cross-linking (Fig. 2D, top panel). In addition, we observed a ubiquitin-dependent increase in molecular weight for the isolated Heimdallarchaeotal UEV (Fig. 2A, green), indicative of direct ubiquitin binding by this domain, rather than a different part of the full-length Vps23-Vps28 protein (Fig. 2D, bottom panel). In eukaryotes, binding is mediated by an isoleucine residue at position 44 (I44) of ubiquitin, which is part of a hydrophobic patch^13^. A model of Heimdallarchaeotal ubiquitin was superimposed on an available crystal structure of ubiquitin in a complex with UEV (Fig. 2B, C), to identify the equivalent residue (V45 in Heimdallarchaeotal ubiquitin). When tested experimentally, the V45D mutation dramatically reduced the interaction between ubiquitin and the UEV domain (Fig. 2D), implying that this interaction resembles the one seen in eukaryotes.

The same was true of the equivalent Odinarchaeotal proteins. When Odinarchaeotal homologues of ubiquitin, Vps23 and Vps28 were purified and mixed in vitro, we observed the formation of a stable complex by size-exclusion chromatography (Fig. 2E), mediated by an interaction between ubiquitin and the UEV domain-containing Vps23 (Fig. 2E, S9, and S10A). Furthermore, when analysed by size-exclusion chromatography, Vps23 alone migrated through the column faster than expected for a monomer (Supplementary Fig. 9). Follow-up size-exclusion chromatography coupled with multi-angle light scattering (SEC-MALS) analyses (Supplementary Fig. 10 A–C) revealed that the Vps23 protein forms a stable dimeric assembly (60.37 kDa), while the calculated mass of the Vps23-Vps28 complex was consistent with a single subunit of Vps28 associating with the Vps23 dimer (yielding a combined molecular weight of 88.27 kDa). In this, the trimeric Odinarchaeotal Vps23-Vps28 complex appears similar to the eukaryotic ESCRT-I subcomplex, which assembles into a heterotrimeric core ‘headpiece’ comprised of Vps23, Vps28 and Vps37^76–78^. As Asgard archaeal genomes encode Vps23-like proteins but lack their Vps37 homologues (Fig. 1A), it is likely that the eukaryotic ESCRT-I (Fig. S8) arose from a heterotrimeric archaeal ESCRT-I subcomplex composed of a Vps23 homodimer and Vps28.

### Structure of the Asgard ESCRT-II subcomplex

**Fig. 3:**
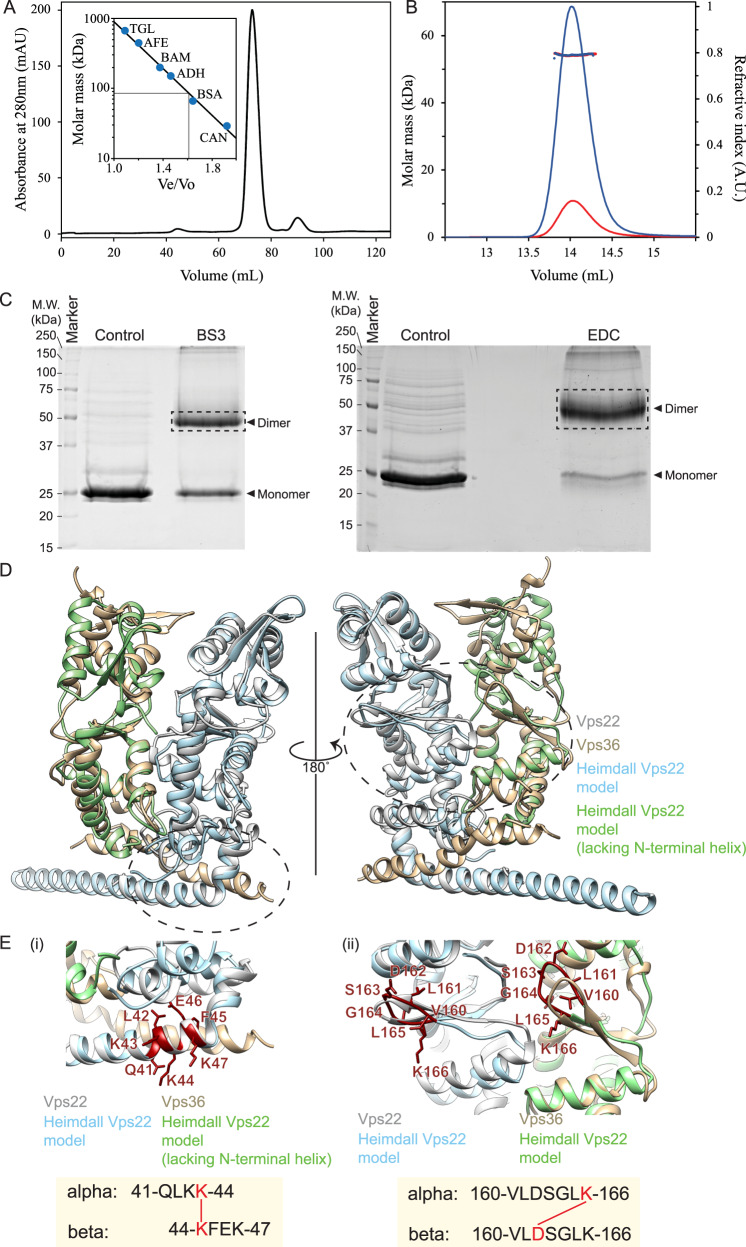
Heimdall Vps22 forms stable dimers. **A** Elution chromatogram of Vps22 (27.9 kDa) using a Superdex 200 16/600 size-exclusion column. The inset shows the column calibration curve established with standard proteins (see Methods section). Grey lines indicate the Ve/Vo and predicted molar mass (85 kDa) of Vps22. This assay suggests that this protein forms a trimer or an elongated dimer. Source data are provided as a Source Data file. **B** SEC-MALS analysis of Heimdall Vps22 using a Superdex 200 increase 10/300 analytical column. The chromatograms display the calculated molar mass of the peaks (kDa) and refractive indexes (A.U.) as dots and lines, respectively, for loaded sample concentrations of 2.0 (blue) and 0.5 (red) mg/ml. The estimated masses are 54.4 and 54.2 kDa for the two protein concentrations, indicating stable formation of a Vps22 dimer, as the theoretical dimer mass is 55.9 kDa. Source data are provided as a Source Data file. **C** Purified HeimAB125 Vps22 showed slower migration on SDS-PAGE gel after chemical crosslinking, whose mobility is consistent with that of a cross-linked dimer. The left panel shows Vps22 treated with or without BS3. The right panel shows Vps22 treated with or without cross-linker EDC. Note that following crosslinking, Vps22 showed a reduced mobility by SDS-PAGE with an estimated molecular weight double of that predicted for monomeric Vps22. Three experimental repeats performed with representative experiment displayed. Source data are provided as a Source Data file. **D** A model structure of HeimAB125 Vps22 superimposed on human Vps22 in the structure of ESCRT-II complex (3CUQ). **E** Vps22-Vps22 interacting regions (i) aa 41-47 (ii) aa 160-166 on the enlarged Vps22 model structure are shown. The crosslink positions on the peptide sequence are highlighted in red in the model structure. The bottom panel shows the cross-linked peptide sequences and the location of the crosslink.

Turning to the ESCRT-II subcomplexes, our genomic analysis identified close homologues of Vps22 and Vps25 in Asgard archaeal genome assemblies, but not full-length Vps36 homologues. Since eukaryotic Vps22 and Vps36 are structurally related^79,80^, and form a Vps22-Vps36 heterodimer, we investigated whether Asgard Vps22 homologues might homodimerize. To test this idea, we used size-exclusion chromatography (Fig. 3A) and SEC-MALS (Fig. 3B) to show that a Heimdall-Vps22 homologue (HeimAB125_14050) migrates at a size consistent with it forming a dimer. Moreover, after chemical crosslinking to stabilize the Vps22 complex, through the addition of BS3 (a homo-bifunctional cross-linker) or EDC (hetero-bifunctional cross-linker that generates zero-length isopeptide bonds), the Vps22 protein band detected using SDS-PAGE had a molecular weight of about 50 kDa, corresponding to a dimer (Fig. 3C). Finally, using chemical crosslinking coupled with mass spectrometry (XL-MS), we identified two dimerization surfaces (41–47 and 160–167 amino acid regions) in the HeimAB125_14050 Vps22 homodimer (Fig. 3D, E and Supplementary Fig. 11). Taken together, these data strongly suggest that Heimdallarcheota AB125 Vps22 forms a homodimer. In light of the phylogenetic analyses (Fig. 1D and Supplementary Fig. 4), it appears likely that the eukaryotic Vps22-Vps36 heterodimer arose during eukaryotic evolution following a gene duplication and diversification event – just as seems to have been the case for the ESCRT-I complex. It is notable, however, that when a SEC-MALS investigation was performed with the Odinarchaeota Vps22, we did not find evidence for its homodimerization. Since the Odinarchaeotal protein was observed to be monomeric (Supplementary Fig. 10D), it is currently unclear if it adopts a homodimeric architecture in the native host conditions or might instead form a heterodimer by interacting with an as-yet undiscovered protein.

**Fig. 4:**
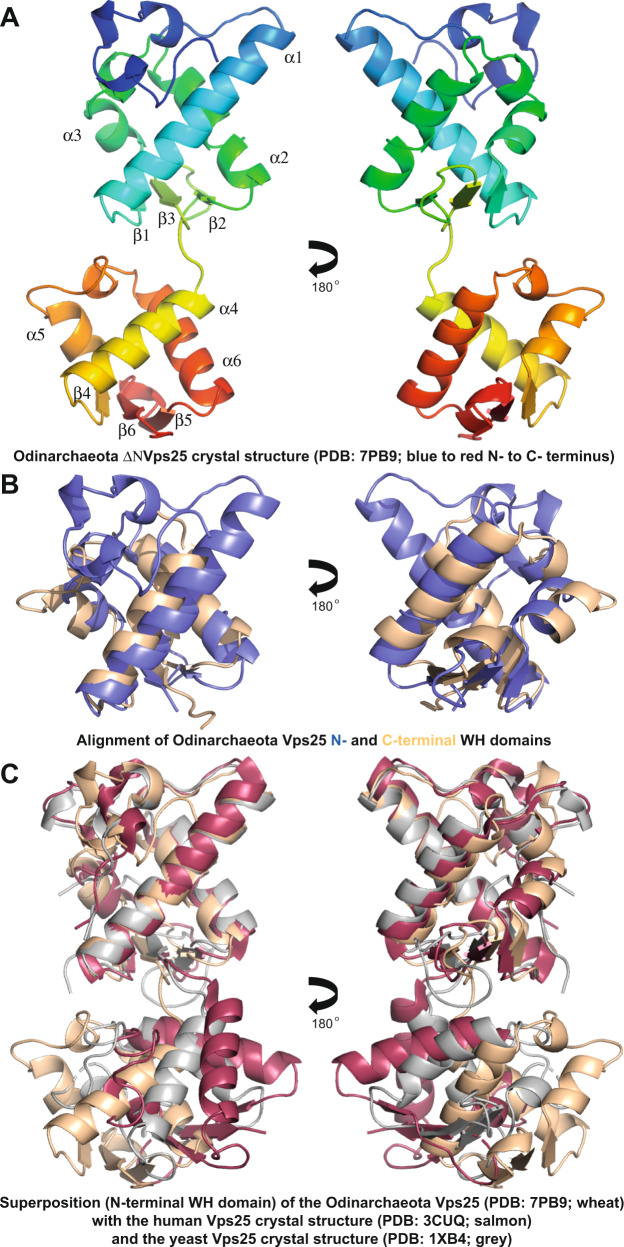
Crystal structure of the Odinarchaeota Vps25ΔN tandem winged helix (WH) domain. **A** Asgard Odinarchaeota Vps25ΔN tandem WH domain structure coloured from blue to red (N-terminus to C-terminus) shown in ribbon form, with secondary structural sequence elements indicated. Refinement and model statistics are shown in Supplementary Table 1. **B** Superposition of the N- (blue) and C-terminal (wheat) Asgard Vps25 WH domains demonstrating their similarity. **C** Structural alignment of the Odinarchaeota Vps25ΔN crystal structure (wheat) with Vps25 from *S. cerevisiae* (grey) (PDB: 1XB4, chain A [https://www.rcsb.org/structure/1XB4]) and *H. sapiens* (salmon) (PDB: 3CUQ, chain C [https://www.rcsb.org/structure/3CUQ]). The alignment shown superimposes the N-terminal WH domains, only, and yields all-atom RMSD values against the Odin N-terminal WH domain of 0.7 Å and 1.1 Å for the yeast and human N-terminal WH domains, respectively. A similar alignment of the C-terminal WH domains yielded all-atom RMSD values of 1.5 Å and 1.1 Å for the yeast and human domains against the Odin C-terminal WH domain. Because of changes in the relative orientations of the WH domains with respect to each other in the tandem arrangement of the three Vps25 homologues, overall alignment gives much higher all-atom RMSD values of 3.5 Å and 5.1 for the yeast and human proteins against Odin, respectively.

Structural models for the Odinarchaeotal and Hemidallarchaeotal ESCRT-II subcomplex proteins generated using AlphaFold 2^74,75^ (Supplementary Fig. 13A, B, C and Supplementary Fig. 14A, B) identified potential tandem winged helix (WH) domains, which they likely share with their eukaryotic counterparts, Vps22 and Vps25^79–81^. We also used CD spectroscopy to confirm that both recombinant Odinarchaeota Vps22 and Vps25 proteins appear well-folded and possess the predicted secondary structural elements (Supplementary Fig. 13 D and Supplementary Fig. 14C). Furthermore, CD thermomelt spectra analyses of these Odinarchaeota ESCRT-II proteins revealed that these proteins are thermostable and remain folded at 60 °C (Supplementary Fig. 13 E and Supplementary Fig. 14D). To further characterise Asgard ESCRT-II subunits, these proteins were then submitted to crystallization trials. In the case of Odinarchaeotal Vps25, while we were unable to crystallize the full-length soluble protein, high-quality crystals could be generated using an N-terminally truncated version (deleting N-terminal residues 1–58), which were then used to determine the structure at a resolution of 1.80 Å (Supplementary Table 1 and Fig. 4A-C). Inspection of this structure revealed that the Asgard Vps25 core is composed of a tandem WH domain repeat, consistent with the predicted AlphaFold 2 model (Supplementary Fig. 13A). The similarity of the Odinarchaeotal Vps25ΔN structure to the yeast and human Vps25 structures (PDBs: 1XB4^82^, chain A [https://www.rcsb.org/structure/1XB4] and 3CUQ^81^, chain C [https://www.rcsb.org/structure/3CUQ], respectively) was quantitatively assessed by all-atom alignment in PyMOL^83^, giving RMSD values of 3.5 Å and 5.1 Å, respectively (Fig. 4C). When doing the same analysis comparing the two WH domains in each structure separately, much smaller all-atom RMSD values indicating strong similarity of 0.7 Å and 1.5 Å are obtained between the N- and C-terminal WH domains of Odinarchaeota Vps25ΔN and yeast Vps25 (PDB 1XB4, chain A [https://www.rcsb.org/structure/1XB4]). This indicates that the two WH domains have significantly different relative orientations, with respect to one another, but are very closely related between organisms when analysed separately. The same single-WH domain analysis against human Vps25 (PDB 3CUQ^81^, chain C [https://www.rcsb.org/structure/3CUQ]) revealed equally low all-atom RMSD values of 1.1 Å and 1.1 Å, respectively, again comparing the N- and C-terminal WH domains separately. Taken together, these findings provide further support for the idea that all the ESCRT-II subcomplex proteins, in eukaryotes and Asgard archaea alike, share a common molecular architecture based on a core tandem WH domain^79,80^. These data reinforce the concept that the ESCRT-II complex arose during archaeal and eukaryotic evolution through a series of gene duplication and specialization events^84,85^.

### Key ESCRT complex protein-protein interactions revealed by yeast two-hybrid analyses of the Asgard archaeal systems

**Fig. 5:**
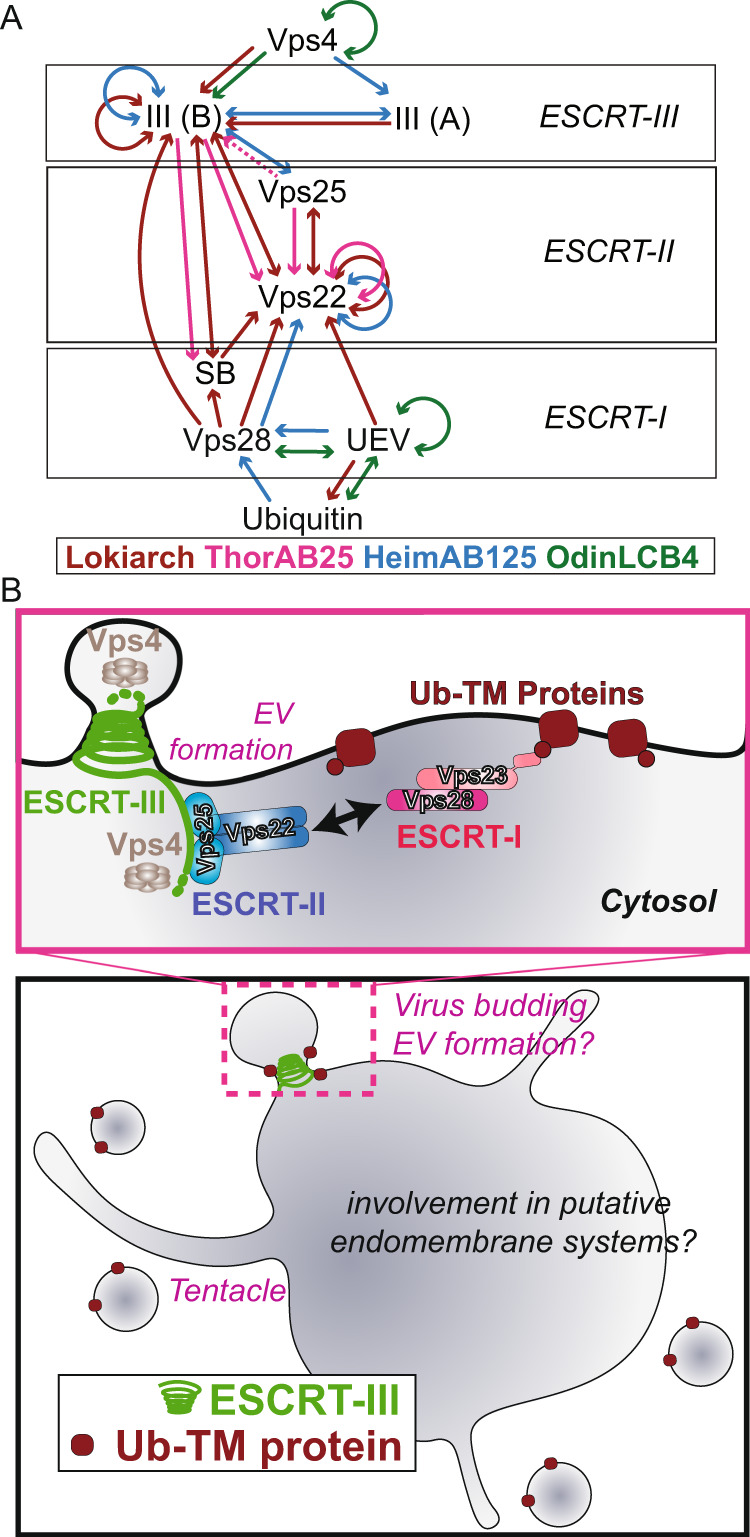
Systematic reciprocal Yeast two-hybrid assays between Asgard ESCRT proteins and insights gained from investigating ESCRT from Asgard. **A** Summary of Y2H interactions. Molecules related to the Ub-ESCRT pathway found in Lokiarchaeota (Lokiarch), Heimdallarchaeota (HeimAB125), Thorarchaeota, and *Ca*. Odinarchaeum yellowstonii LCB_4 (OdinLCB_4) were examined comprehensively using Y2H, and the detected interactions are illustrated. See Figs. 3, 4 and S9 for further biochemical validations. **B** Top panel: Schematic representation of the organization of the Asgard ESCRT pathway based on this work. In the Heimdallarcheota and Lokiarchaeota ESCRT-II complexes, the Vps22 subunit forms a homodimer like the eukaryotic Vps22/Vps36 ESCRT-II heterodimeric stalk. In Odinarcheota, however, the Vps22 homologue does not appear to dimerise, and yet undetermined factor(s) therefore likely bridge the interaction between the ESCRT-I and -II subcomplexes. The Odinarcheota Vps23 ESCRT-II homologue forms a dimer, thereby presenting two ubiquitin-binding UEV domains. The Vps23 dimer interacts with a single Vps28 protein thus forming a tripartite complex, reminiscent of the eukaryotic Vps37/Vps23/Vps28 complex. In Heimdallarcheota, the Vps23 and Vps28 functions are fused in a single protein that also dimerises. Compare with the eukaryotic arrangement as shown in Supplementary Fig. 1. Bottom panel: a schematic representation of a hypothetical Asgard archaeal cell using ESCRT-III polymers to facilitate extracellular vesicle formation and potentially in virus release.

Since the ESCRT-I and ESCRT-II systems characterized above function as potential protein bridges that physically connect Ub-modified proteins with the ESCRT-III machinery, we needed a systematic way to sensitively test for interactions within and across different subcomplexes. To do so, we carried out a comprehensive reciprocal yeast two-hybrid analysis (Y2H) in budding yeast to identify pairwise protein interactions for the full set of Ub-ESCRT homologues from Thor-, Odin-, Loki- and Heimdallarchaeota (Fig. 5A). As a control for this analysis, we used the same approach to systematically probe for interactions between proteins that are known to function as part of the ESCRT system in the fission yeast *Schizosaccharomyces pombe* (Supplementary Fig. 15 and Supplementary Fig. 16). Importantly, in these control experiments, we were able to identify many of the expected interactions between components of the Ub-ESCRT system in fission yeast. This included the previously reported interactions within the respective ESCRT complexes; ESCRT-I (Sst6-Vps28), ESCRT-II (Vps22-Vps25, Vps36-Vps25), ESCRT-III (Vps20-Vps32, Vps24-Did4), as well as published interactions that bridge the eukaryotic ESCRT-I and -II subcomplexes (Vps28-Vps36) and those that connect ESCRT-II and -III (Vps20-Vps25, Vps20-Vps22) (Supplementary Fig. 13A).

We then applied Y2H analyses to systematically search for protein-protein interactions between ESCRT-related components encoded by Asgard archaeal genomes (Fig. 5A). As expected, these assays revealed interactions between ESCRT-III components and the Vps4 ATPase. In line with the biochemical data presented above, these Y2H analyses also identified interactions between ubiquitin and UEV domain-containing proteins of Heimdallarchaeota, Odinarchaeota and Lokiarchaeota (Fig. 5A and S14). Furthermore, the UEV domain-containing Vps23 homologues from Odinarchaeota and Lokiarchaeota displayed interactions with Vps28, as expected if they formed an ESCRT-I subcomplex, whereas the Heimdallarchaeota possess a Vps23-Vps28 fusion protein homologue discussed above. The Y2H assays also detected the interaction between ubiquitin and the Heimdallarchaeotal ESCRT-I fusion protein (Fig. 5A and Supplementary Fig. 15B). Interestingly, this analysis also suggested an alternative pattern of protein interactions for the corresponding ESCRT-I proteins from Lokiarchaeota and Thorarchaeota. In these two cases, the freestanding steadiness box protein, equivalent to the alpha-helical hairpin ‘headpiece’^72,76,86^ coded within the same genomic neighbourhood as the rest of the Lokiarchaeotal ESCRT machinery (Fig. 1C), may interact with Vps28 – mirroring the role of its eukaryotic counterpart in mediating interactions between Vps23, Vps28 and Vps37.

The Y2H analyses also identified multiple interactions between the Lokiarchaeota and Heimdallarchaeota ESCRT-I (Vps28 and/or Vps23) and ESCRT-II (Vps22) complexes. In addition, the Y2H experiments indicate that the ESCRT-II component Vps22 from Heimdall-, Loki- and Thorarchaeota can interact with themselves, in agreement with the biochemical assays shown above, suggesting the formation of homodimers. Finally, we identified numerous interactions linking the Asgard ESCRT-II and -III (Vps25-ESCRT-IIIB), Vps4-ESCRT-III and between the ESCRT-III homologues (-IIIA and -IIIB) (Fig. 5A and Supplementary Fig. 16). This analysis also suggests the possibility of additional interactions between ESCRT-I and II subcomplexes with ESCRT-III proteins that bypass Vps25 in Loki- and Thorarchaeota. We note that although we were unable to detect many such interactions between the Odinarchaeotal proteins using the Y2H approach, *Ca*. Odinarchaeum yellowstonii LCB_4 is a thermophile. Thus, it seems reasonable to suggest that the temperature used in these experiments (25 °C) may have negatively affected our ability to identify interactions between proteins that are optimized to fold and work at much higher physiological temperatures^58^.

## Discussion

Here we provide experimental and computational support for the idea that many Asgard archaea possess a streamlined version of the Ub-ESCRT system present in eukaryotes (Fig. 5B). It is clear from our analyses, however, that the precise composition differs between species and across phyla. This is especially the case for ESCRT-I subunit architectures. However, with the exception of Thorarchaeota, in which ubiquitin encoding genes are yet to be identified, we identified ubiquitin-binding UEV-domain proteins in all the genomes analysed. While these domains were often harboured within proteins homologous to Vps23, which include a C-terminal alpha-helical headpiece region involved in ESCRT-I complex assembly, in other systems this alpha-helical ‘steadiness box’ domain was encoded by a freestanding protein. Alternative ESCRT-I domain arrangements were also observed, such as the UEV-Vps23-Vps28 fusion found in Heimdallarchaeota (Supplementary Fig. 8). Taken together, the clear synteny between genes of the ubiquitylation apparatus and the ESCRT machinery in the Loki-, Hel-, Odin-, and Heimdallarchaeotal genomes, and the experimentally verified Y2H interactions between ubiquitin and UEVs in all the Asgard archaea investigated, support a model in which ubiquitylated substrates recruit the ESCRT-I in Asgard archaea.

In line with this, we were able to demonstrate direct binding between ubiquitin and UEV-containing proteins that was dependent on a conserved hydrophobic patch in ubiquitin. Although Asgard archaea appear to lack homologues of the eukaryotic ESCRT-I subunit Vps37 (which assembles into a heterotrimer with Vps23 and Vps28), it is notable that Vps23 and Vps28 from Odinarchaeota assemble into a similar trimer that contains two copies of Vps23 instead. It is therefore possible that the three proteins of the eukaryotic ESCRT-I complex evolved from a common ancestor containing an alpha-helical hairpin region; with Vps37 having arisen as a eukaryotic innovation^72^.

Eukaryotic ESCRT-II forms a ‘Y-shaped’ hetero-tetrameric structure consisting of a Vps22/Vps36 stalk which binds two Vps25 subunits^79,80^. Although Vps22 and Vps25 coding genes were readily identifiable in the Asgard archaeal genomes, as reported previously^57,58^, we were unable to identify Vps36 homologues. However, as with ESCRT-I, our biochemical and Y2H interrogation suggest that the Asgard ESCRT-II proteins from several Asgard phyla function together in a structurally similar manner to their eukaryotic counterparts, with the formation of Vps22 homodimers making up for the lack of Vps36. In the corresponding eukaryotic complex, all three of these ESCRT-II proteins (Vps22, Vps36 and Vps25) contain an evolutionarily conserved globular core consisting of tandem winged helix (WH) domains^79–81^. The same appears to be true for the Asgard ESCRT-II machinery which, based upon modelling of Vps22 and Vps25 proteins from Odinarchaeota and the crystallographic Odinarchaeotal Vps25 structure, possess two very similar WH domains. This suggests that all ESCRT-II proteins were initially derived from a single-WH domain protein progenitor, with Vps36 emerging during eukaryogenesis.

Do ESCRT-I, -II, and -III contribute to a single pathway? The genomic colocation and synteny analyses strongly suggest this possibility. The co-location of ESCRT-III with Vps25 indicates that these proteins likely function in a related biochemical process. Y2H experiments revealed consistent interactions between Vps25 and ESCRT-IIIB. Furthermore, this analysis provided support for there being an interaction between ESCRT-I and ESCRT-II. We also found an interaction between the Lokiarchaeota steadiness box protein and Vps22, supporting the existence of physical interactions between the ESCRT-I and ESCRT-II subcomplexes. These Y2H data and the corresponding gene cluster analysis point to ESCRT -I, -II, and -III functioning in concert in Asgard archaea – although definitive confirmation of this will require future studies using Asgard archaeal cells and cell extracts. While it is not yet clear how the Ub-ESCRT system evolved, we note that whereas ESCRT-III superfamily proteins can be traced back to the last universal common ancestor (LUCA)^64^, the Vps4 and ESCRT-III pair can only be found in archaea, whereas Ub, ESCRT-I and ESCRT-II components are only found *together* in Asgard archaea (with the exception of Thorarchaeota, which seemingly lack ubiquitin homologues). This suggests a plausible pathway for the stepwise evolution of the eukaryotic ubiquitin-directed ESCRT-dependent membrane trafficking system. From simple beginnings in an archaeal progenitor, the machinery grew in complexity through successive rounds of domain concatenation, gene duplication and divergence (Fig. 5B).

Although no evidence has been found to date to suggest that the only cultured member of the Asgard superphylum, *Prometheoarchaeum syntrophicum*, possesses an endomembrane system^87^ we do not yet know if endomembrane compartmentalization has arisen in the diverse and ever-expanding Asgard superphylum. Significantly, a rudimentary endomembrane system has already been identified in a TACK archaeon, *Ignicoccus hospitalis*^88^. It has also been reported that crenarchaeal species generate extracellular vesicles that are dependent on ESCRT-III function^89,90^. The ESCRT-III machinery of crenarchaeal model systems has been shown to be essential for cell division^91,56^ and also for the egress of the turreted icosahedral virus (STIV)^90,92^. It is therefore plausible that ESCRT-III homologues in Asgard archaea may similarly participate in a variety of membrane-sculpting and scission events. The archaeal origins of eukaryotic ESCRT-III-mediated membrane remodelling has been well documented^56,91,93–96^. Our characterization of a eukaryotic-like ubiquitin-linked ESCRT-I subcomplex and associated ESCRT-II subcomplex components further suggests that the earliest steps in eukaryotic membrane remodelling evolved from a simpler Asgard archaeal system. Indeed, it is possible that the Ub-ESCRT membrane remodelling machinery already present in the last common ancestor of Asgard archaea and eukaryotes played a role in the elaboration of membranes required for eukaryogenesis itself^97^. Unravelling the precise cell biological functions and evolutionary significance of the Ub-ESCRT systems will require detailed cell biological analyses of representatives of the Asgard superphylum—a tantalising prospect for the years to come.

## Methods

### Genomic survey of protein homologues

All genomes from organisms classified as Asgard archaea were downloaded from NCBI on December 5^th^, 2020. These genomes were taxonomically reclassified through a phylogenetic analysis based on a set of 15 ribosomal proteins encoded in co-locating genes^98^. To ensure annotation homogeneity, protein sequences were predicted de novo using Prodigal v2.6.3^99^, and ribosomal protein genes were detected using psiblast^100^ using predetermined orthologous sequences^58^, aligned with Mafft-linsi v7.450^101^ and processed with trimAl v1.4.rev22^102^ to remove sites with over 50% gaps. All genomes containing at least 5 of these proteins were concatenated and used to reconstruct a tree with IQ-Tree v2.0-rc1^103^ the LG + C60 + R4 + F model, using 1000 pseudoreplicates for ultrafast bootstrap^104^ and SH-approximate likelihood ratio tests.

These reclassified genomes were then annotated using InterProScan v5.48-83.0^105^. A set of Interpro domains were used as diagnostic for the inference of Vps23/37 (IPR017916), Vps28 (IPR007143, IPR037206), Vps23/37/28 (IPR037202), Vps22/36 (IPR016689, IPR040608, IPR021648), Vps25 (IPR008570, IPR014041), ESCRT-III (IPR005024), Vps4 (IPR007330, IPR031255, IPR015415), ubiquitin (IPR029071, IPR000626), ubiquitin-activating enzyme E1 (IPR000594), ubiquitin-conjugating enzyme E2 (IPR000608, IPR006575, IPR016135), ubiquitin-ligase enzyme E3 (IPR018611), and deubiquitinating enzyme (IPR000555) genes.

Co-location of these genes was investigated through custom perl scripts and visualized using R^106^ and the packages ggplot2^107^, cowplot^108^, and genoPlotR^109^.

### Generation of model structures using AlphaFold 2

#### Heimdallarchaeotal Vps25 structural model

HeimAB125_14040 full amino acid sequence was used for structural modelling with Alphafold 2 server (https://colab.research.google.com/github/sokrypton/ColabFold/blob/main/AlphaFold2.ipynb). This server uses MMseqs2 and HHsearch^75^. The top-ranked model was used for the structural analysis. The first 80 amino acid region did not show high IDDT score so was removed from the model. A region from the 177th amino acid residues to the C-terminus of the model was superimposed on an available crystal structure of the Vps25-Vps20 complex (PDB: 3HTU^110^ [https://www.rcsb.org/structure/3HTU]). The 81:178 amino acid region of the model was superimposed on Vps25 in the crystal structure of the ESCRT-II core protein complex (PDB: 3CUQ^81^ [https://www.rcsb.org/structure/3CUQ]).

#### Heimdallarchaeotal Vps28 domain structural model

Amino acid residues 278–466 of HeimAB125_14070 were used for structural modelling with Alphafold 2 server as described above. The model structure was superimposed on the crystal structure of human ESCRT-I headpiece, consisting of Vps37/Tsg101/Vps28 (PDB: 6VME^78^ [https://www.rcsb.org/structure/6VME]) as well as Vps28/Vps36 (PDB: 2J9U^111^ [https://www.rcsb.org/structure/2J9U]).

#### Structural model of Heimdallarchaeotal Vps23/Tsg101 ESCRT-I core region

113:277 residues of HeimAB125_14070 were used for structural modelling as described above. The top-ranked structure predicted a long N-terminal helix at 150:277, which did not superimpose well with Tsg101. On the contrarily, the second-ranked structure showed a split helix around 149:190 and 191:277. The latter showed good superimposition with the Tsg101 (Vps23) headpiece (PDB: 6VME^78^ [https://www.rcsb.org/structure/6VME]). The 149:190 helix could be either superimposed with the 1st or 2nd helix of Vps37 (6VME^78^ [https://www.rcsb.org/structure/6VME]).

#### Heimdallarchaeotal UEV and UBC model

Residues comprising the UEV or UBC domain of HeimAB125_14070 (24:112aa), HeimAB125_07740 (6:146aa), HeimAB125_09840 (1:118aa) and HeimAB125_11700 (full-length) were used for structural modelling as described above. The 91 to C-terminus region of the HeimAB125_11700 was deleted owing to low IDDT scores. Resulting models were superimposed with the crystal structure of ubiquitin-conjugating enzyme E2 ligase (PDB: 1JBB^112^ [https://www.rcsb.org/structure/1JBB]).

#### Heimdallarchaeotal Vps22 structural model

All residues comprising HeimAB125_14040 were used for structural modelling as described above. The resulting model seemed reliable within the helical N-terminal part (aa 1:81) and within the rest of the core fold (aa 82 to C-terminus). These regions were individually superimposed on the crystal structure of Vps22 in the ESCRT-II core complex (PDB: 3CUQ^81^ [https://www.rcsb.org/structure/3CUQ]) and on Vps36 in the same structure.

#### Heimdallarchaeotal ubiquitin structural model

HeimAB125_14240 full amino acid sequence was used for structural modelling as described above. The rank1 model structure was used for the analysis. The model structure was superimposed with ubiquitin (PDB: 1UZX^113^, chain B [https://www.rcsb.org/structure/1UZX]).

#### Odinarchaeotal Vps25 AF2 structural model

All residues comprising OdinLCB_4_14300 were used for structural modelling as described above. The model structure was superimposed *on Saccharomyces cerevisiae* Vps25 (PDB: 1XB4^82^, chain A [https://www.rcsb.org/structure/1XB4]).

#### Odinarchaeotal Vps22 AF2 structural model

All residues comprising OdinLCB_4_14290 were used for structural modelling as described above. The model structure was superimposed on *Homo sapiens* Vps22 (PDB: 3CUQ^81^, chain A [https://www.rcsb.org/structure/3CUQ]).

### Asgard archaeal proteins used in this study

The Heimdall-, Loki-, Odin-, and Thorarchaeota amino acid sequences were obtained from Uniprot (https://www.uniprot.org/) and the Uniprot entry IDs are listed in Supplementary Data 1. The corresponding genes were synthesized for the expression in *E. coli* and yeast. The *Ca*. Odinarchaeum yellowstonii LCB_4 ORFs (Uniprot Entry IDs and amino acid sequences are shown in Supplementary Data 1) were PCR amplified form MDA amplified environmental DNA isolated from the Lower Culex Basin Yellowstone National Park, USA as described^114^ and cloned into either pET28a (Vps23 and Vps28) or pET30 (Vps22 and Vps25) (Novagen), respectively. Details of the oligonucleotides used to PCR amplify the ORFs are provided in Supplementary Table 2.

### Plasmids used in this study

The Asgard archaeal genes obtained by gene synthesis were cloned into yeast two-hybrid (Y2H) vectors and *E. coli* expression vector. The oligonucleotides and plasmids for Y2H are listed in Supplementary Tables 2 and 3.

### Systematic, reciprocal yeast two-hybrid assays

Y2H assays were performed using the set of genes listed in Supplementary Table 3. The plasmids used in this study are listed in Supplementary Table 3. Indicated genes of interest were cloned both in “bait-ProteinA” and “prey-ProteinB” vectors or vice versa, which have DNA binding protein LexA and/or activation domain of Gal4p were cloned into pMM5 and pMM6 plasmids respectively^115,116^. Plasmids carrying these constructs were transformed into the yeast strains SGY37 (MATa) and YPH500 (MATα). Transformants with plasmids plexADBD (pMM5) and pGal4AD (pMM6) were selected on plates lacking Histidine or Leucine, respectively. After mating, the two strains carrying the desired plasmids were grown on YPD plates for 2 days at 30 °C and replica plated on selection plates (without Histidine and Leucine) for 2 days at 30 °C before the overlay. The interaction between the protein products fused to the DNA binding and activation domains were analyzed by the activity of β-galactosidase by the cleavage of X-Gal (BIO-37035, Bioline, UK). For detecting the β-galactosidase activity overlaying of low melting agarose with X-Gal (overlay mix was prepared freshly), overlay solution was added slowly on to the plates. Interaction of LexA-Protein-A with Gal4-Protein-B resulted in the activation of expression of the lacZ gene coding for β-galactosidase, converting X-Gal to produce blue colour. Plates were monitored every 30 min to see the appearance of blue colour. Plates were scanned after 16 hr of incubation with the X-Gal overlay mixture.

### Phylogenetic reconstruction

#### UEV and E2

Amino acid sequences of UEV domain-containing proteins, TSG101/Vps23 and UBC domain-containing proteins in *H. sapiens*, *S. cerevisiae*, *D. discoideum*, *E. histolica*, *A. thaliana*, *C. marolae*, *T. brucei*, *T. pseudonana* and *T. parva* were obtained from Uniprot. Asgard E2L proteins from *Ca*. Odinarchaeum yellowstonii LCB_4, Heimdallarchaeota (strains AB125, LC2 and LC3), and Lokiarchaeota (strains GC14_75 and *CR*_4) were also obtained from Uniprot. These sequences were aligned with Mafft-linsi v7.450, and the resulting multiple-sequence alignment was used as query for a Psiblast (v2.10.0+) against all Asgard archaeal genomes (see Genome survey of protein homologues). All hits with e-values lower than 1e-5 were used together with query sequences and aligned using Mafft-linsi. The resulting alignment was trimmed using trimAl v1.4.rev22, and sequences containing over 60% gaps in the trimmed alignment were removed. The obtained alignment was used for a phylogenetic reconstruction with IQ-Tree 2.0-rc2^103^, under the model Q.pfam + C20 + G4 + F, chosen by ModelFinder^117^ between combinations of empirical matrices (LG, WAG, JTT, and Q.pfam) with mixture models (C20, C40, and C60) and various rate heterogeneity (none, G4 and R4) and frequency (none, and F) and using 1000 ultrafast bootstrap pseudoreplicates. The resulting phylogeny was used as guide to reconstruct another tree under the PMSF approximation of the chosen model and using 100 non-parametric bootstrap pseudoreplicates. The resulting bootstrap trees were used both using the standard Felsenstein Bootstrap Proportion and the more recent Transfer Bootstrap Expectation^118^ interpretations.

#### Vps22 and Vps36

Eukaryotic Vps22, Vps36 and Vps25 and Asgard Vps22/36 and Vps25 homologue sequences were downloaded from NCBI. These 187 sequences were aligned using Mafft-linsi v7.450 and trimmed with trimAl with the parameter “-gappyout”. A maximum-likelihood tree was then reconstructed using IQ-Tree v2.0-rc1 under the model LG + C60 + R4 + F, using 1000 ultrafast bootstrap and SH-approximate likelihood ratio test pseudoreplicates. In parallel, potential outgroup sequences (eukaryotic Rpc35/Rpc6, Asgard archaeal UFM1 and bacterial ScpB) were downloaded and added to the previous sequences. Three additional Asgard archaeal sequences were found to contain potential plekstrin domains and were used as query for a Blast-p search against the Asgard archaeal proteomes to recruit homologues identified as hits with e-values lower than 1e-10. The resulting set of 314 sequences was then aligned with Mafft-linsi v7.450 and trimmed with trimAl to remove all sites with over 90% gaps. The resulting trimmed alignment was used to reconstruct a maximum-likelihood tree using IQ-Tree v2.0-rc2^103^ under the PMSF approximation^119^ of the LG + C60 + R4 + F model using 100 non-parametric bootstrap pseudoreplicates. The resulting bootstrap trees were used both using the standard Felsenstein Bootstrap Proportion and the more recent Transfer Bootstrap Expectation interpretations. To ensure we did not miss possible homologues of ESCRT-II sequences outside of Asgard archaea, we used the previous set of 187 Vps22/Vps36/Vps25 sequences as query for a psiblast search against the NR database (1 iteration, e-value threshold of 1e-10), and parsed the resulting 4745 hits to remove proteins originating from Asgard archaeal or eukaryotic genomes. After parsing, only nine sequences remained, belonging to various putative archaea and bacteria. A Blast-p search of these sequences against NR confirmed that their best hits were Asgard archaea or eukaryotic sequences. We added these sequences to the previous 227 Vps22/Vps36/Vps25/Outgroup sequences, aligned them with Mafft-linsi v7.450 and trimmed with trimAl to remove sites with over 50% gaps. We used this alignment to reconstruct a tree with IQ-Tree under the LG + C20 + G4 + F model, using 1000 ultrafast bootstrap and SH-approximate likelihood ratio test pseudoreplicates. The resulting tree confirmed that these nine homologues were well embedded in the clades of Asgard archaeal or eukaryotic Vps22, thus likely representing Asgard archaeal or eukaryotic Vps22 sequences that have been misclassified in public databases.

### Protein purification

#### Heimdallarchaeotal protein expression and purification

The vector carrying a 6-His residues (His-tag) followed by SUMO protein from *Brachypodium distachyon* were generated as described before^120,121^ with slight modification. The gene of *B. distachyon* SUMO protein (bdSUMO) was synthesized (IDT gBlock) and cloned into pET28a in frame with sequence encoding the N-terminal His-tag. The bdSUMO sequence was codon optimized for the expression in *E. coli* K12 strain. The resulting vector, pSUMO was used as the backbone for cloning the Heimdallarchaeotal ESCRT genes for their expression as SUMO-tag fusions in *E. coli* BL21(DE3). To express and purify SUMO protease in *B. distachyo*, the *B. distachyo* SENP1 gene was synthesized with codon optimization for expression in *E. coli* (IDT gBlock). The gene fragment was cloned into pET28a vector in-frame with N-terminal His-tag. His-bdSENP1 was expressed in BL21(DE3) and purified and used for SUMO-TAG cleavage.

Untagged Heimdallarchaeotal proteins were expressed as N-terminal His-SUMO fusions (His-SUMO) from the pSUMO vector. After the affinity purification using His-Nickel interaction, the His-SUMO was cleaved by His-bdSENP1 and both the cleaved N-terminal His-SUMO tag and His-bdSENP1 were absorbed on a Ni-NTA column. The untagged recombinant protein was further purified by size-exclusion chromatography (SEC). Details of the oligonucleotides used to PCR amplify the Heimdallarchaeotal ORFs are provided in Supplementary Table 2.

Heimdallarchaeotal Vps22 and Full-length or UEV-domain of Heimdallarchaeotal UEV-Vps28 (HeimAB125_14070) and Ubiquitin (with an N-terminal His tag) proteins were expressed in *E. coli*. Cells were grown to an OD_600_ of 0.6 and induced overnight with 0.33 mM IPTG at 20 °C. After protein expression the cells were resuspend in lysis buffer (50 mM Tris-HCl (pH 7.5), 2.5 mM MgCl2, 150 mM NaCl, 2 mM DTT, 2 mM ATP, and 15 mM Imidazole), containing 2x concentration of PIC (Roche complete, EDTA-free #05056489001) and 2 mM PMSF. Lysis was achieved using a pressure homogenizer (Stanstead #FPG12800, 20-30 psi, several passes with solution precooled to 5 °C). The proteins were then affinity purified by incubating with 2 ml Ni-NTA resin (Thermo #88222) for 1 h at 4 °C. The resin was washed with 200 mL ice-cold lysis buffer, followed by 150 ml ESCRT-buffer (50 mM Tris-HCl (pH 7.5), 2.5 mM MgCl2 and 150 mM NaCl). The bound protein was eluted with ESCRT-buffer containing 300 mM imidazole. Elution fractions were combined and concentrated to a volume of 500 µl. The sample was spun at 21000 × *g* for 15 min at 4 °C and the supernatant was applied to 16/60 sephacryl S-100 HR column (GE Healthcare) equilibrated with ESCRT-buffer. Appropriate fractions from the SEC were concentrated followed by high-speed centrifugation at 4 °C (21000 × *g*, 15 min) to remove any insoluble material. The samples were snap-frozen and stored at −80 °C.

#### Odinarchaea protein expression and purification

Thermophilic Odinarchaeota proteins were expressed in Rosetta (DE3) pLysS *Escherichia coli* cells (Novagen). PCR amplified genes were cloned into expression plasmids (pET28a or pET309b) using *Nde*I and *Xho*I restriction sites placing the ORFs in frame with the plasmid-encoded hexa-histidine tags. (Details of the oligonucleotides used to PCR amplify the ORFs are provided in Supplementary Table 2).

Transformed cultures were grown at 37 °C to an OD_600_ of 0.3 then cooled to 20 °C and further grown to an OD_600_ of 0.6 and induced overnight with 0.33 mM IPTG. Cells expressing the recombinant Odinarchaeota proteins were harvested by centrifugation, resuspended in 20 mM Tris-HCl (pH 8.0), 300 mM NaCl, 5% glycerol, 0.05% β-mercaptoethanol. 1X EDTA-free protease inhibitors (Roche complete, EDTA-free) were added and cells were lysed by sonication and heat clarified at 60 °C for 20 min before centrifugation at (23 708 *g* for 10 min) to remove insoluble material. Supernatants were filtered and then purified by IMAC by gravity flow to a column of Ni-NTA agarose (Qiagen). The columns were washed with resuspension buffer and then resuspension buffer plus 15 mM imidazole. Proteins were then eluted in resuspension buffer plus 500 mM imidazole. Fractions containing the purified proteins were pooled and concentrated before running a size-exclusion chromatography (SEC) step over a Superdex 200 16/600 column (GE Healthcare), in 20 mM Tris-HCl pH 8, 300 mM NaCl, 5% glycerol, 0.5 mM dithiothreitol. N-terminal His-tags were then removed from the Odinarchaeota Vps23(TSG101) and Vps28 proteins by thrombin cleavage and further purification by SEC. Fractions containing the purified proteins were pooled, concentrated, aliquoted and flash-frozen in liquid N_2_. Protein concentrations were quantified by UV spectrophotometry.

#### Analytical size-exclusion chromatography

Heimdallarchaeota Vps22 (27.9 kDa) was subjected to analytical SEC using a Superdex 200 16/600 size-exclusion column (GE Healthcare). The sample was loaded onto the column in a buffer comprised of 20 mM Tris-HCl pH 8.0, 200 mM NaCl and 5% (v/v) glycerol at a flow rate of 0.5 mL/min. The calibration curve was established under the same conditions using the following standard proteins (Sigma MWGF1000): carbonic anhydrase (CAN; 29 kDa), bovine serum albumin (BSA; 66 kDa), alcohol dehydrogenase (ADH; 150 kDa), beta-amylase (BAM; 200 kDa), apoferritin (AFE; 443 kDa) and thyroglobulin (TGL; 669 kDa). Physical interactions between the Odinarchaeota ESCRT-I complex proteins (Vps23, Vps28 and ubiquitin) were examined by size-exclusion chromatography using an analytical Superdex S200 HR 10/300 column (GE Healthcare). Prior to the gel filtration analyses, ESCRT-I complexes were formed at 60 °C by mixing 250 μg of each protein in a final volume of 500 μl gel filtration buffer (20 mM Tris [pH 8.0], 150 mM NaCl, 5% glycerol, 1 mM DTT) for 10 min. The complexes were subsequently spun at 16,000 *g* in a benchtop centrifuge for 5 min to remove any precipitated material, before loading onto the size-exclusion chromatography column. 0.5 ml fractions were collected and resolved by SDS-PAGE, on 15% polyacrylamide gels. The proteins were then visualized with Coomassie stain.

#### Size-exclusion chromatography–multi-angle laser light scattering (SEC-MALS)

The molecular mass and oligomeric state of Heimdallarchaeotal Vps22 was determined in solution using SEC-MALS. Data were obtained with a Wyatt HeleosII18 angle light scattering machine connected to a Wyatt Optilab rEX online refractive index detector (Wyatt Technology). Samples were purified using a Superdex 200 increase 10/300 analytical gel filtration column (Cytiva) coupled to an Agilent 1200 series LC system at 0.5 ml/min in 20 mM Tris-HCl pH 8.0, 200 mM NaCl buffer before detecting the light scattering and refractive index in a standard SEC-MALS format. Protein concentration was obtained from the excess differential refractive index of 0.185 ∆RI for 1 g/ml or using the sequence UV extinction coefficient of 0.964 at 280 nm for 1 mg/ml calculated by ProtParam. The determined protein concentration and scattering intensities were used to estimate the molecular mass from the intercept of a Debye plot using Zimm’s model and the Wyatt ASTRA software. The experimental configuration was checked with a BSA standard, run in the same buffer and using the same sample injection volume of 100 uL. The BSA monomer peak was utilized to examine the mass determination and to inspect the inter-detector delay volumes and band broadening parameters that were used during analysis in Wyatt’s ASTRA software. The SEC chromatogram, showing RI as concentration signal, is shown in Fig. 3B as blue and red lines for loaded sample concentration of 2 and 0.5 mg/ml, respectively. The Odinarchaeota Vps22, Vps23, Vps25 and Vps28 proteins were also analysed by SEC-MALS. These data were obtained using a miniDAWN TREOS MALS detector system with a 60 mW laser source at 664 nm, and three fixed angle detectors at 49. 90, and 131 degrees (Wyatt Technology), followed by a Shimadzu RID-20A Refractive Index Detector at 30.5 °C. 100 μl of each protein at 2 mg/ml were passed over a Superdex 200 10/300 Increase GL column (GE Healthcare), in 20 mM Tris (pH 8.0), 300 mM NaCl at 0.4 ml/min. The column output was fed into the detector system. The experimental configuration was calibrated with a BSA standard, run in the identical 20 mM Tris (pH 8.0), 300 mM NaCl buffer using the same 100 uL injection volume. The BSA monomer peak was utilized to examine the mass determination and to normalise the laser and detectors of the light scattering detector and inspect the inter-detector delay volumes and band broadening parameters that were used during analysis in Wyatt’s ASTRA software, with the refractive index increment (dn/dc) set at 0.18 for all samples.

#### Circular dichroism (CD)

Proteins were buffer exchanged into freshly prepared buffer (10 mM potassium phosphate, 50 mM sodium sulphate, pH 7.2) using PD-10 desalting prepacked columns (Sephadex G-25M, GE Healthcare) following manufactures instructions. Theoretical extinction coefficients determined from amino acid sequence (http://www.expasy.ch/tools/protparam.html) were used to estimate protein concentrations for circular dichroism from the absorbance at 280 nm using a Nanodrop spectrophotometer. Protein concentrations were then adjusted to 5 μM using buffer. CD spectra (in triplicate) were acquired using a Chirascan Plus Benchtop CD spectrophotometer over 180–260 nm with a bandwidth of 2 nm and a pathlength of 0.2 cm. The mean buffer subtracted CD spectra (measured ellipticity: mdeg) were interpolated between 190–250 nm using Origin Pro 2018b and fitted to the BeStSel algorithm to determine the secondary structural elements^122^. Structural models for each ESCRT protein were generated using AlphaFold 2^74,75^ after which the STRIDE web server^123^ was used to estimate secondary structure elements for comparison with the CD derived estimations. Thermomelt CD spectra were acquired, as described above in 10 °C increments over 20–90 °C temperature range and a bandwidth of 1 nm. 6 min was allowed for the temperature to settle between each 10 °C increment. The CD thermomelt spectra profiles of Odinarchaeota ESCRT-II proteins remained relatively unchanged below 60 °C suggesting that they are stable, and do not significantly unfold up to this temperature.

#### ΔN_Vps25 crystallization conditions

An N-terminally truncated Vps25 expression construct (removing the first 58 amino acids) was generated using the primers and OdΔN_ESCIIV25forXhoI and OdESCIIV25revXhoI as described in Supplementary Table 2. The protein was purified as described above, except 5.25 mM TCEP was used as the reducing agent in the final size-exclusion chromatography step.

ΔN_Vps25 crystals were grown by sitting-drop vapour diffusion using our in-house high-throughput crystallization platform^124^. Vps25 was used at a concentration of 21.4 mg/ml and the best crystals were obtained in the condition E12 of the Morpheus screen^125^: 120 mM ethylene glycols, 100 mM buffer 3 (26.7 ml 1 M bicine plus 23.3 ml 1 M Trizma base), 12.5% (w/v) PEG 3350, 12.5% (w/v), 12.5% (w/v) PEG 1 K, 12.5% (w/v) MPD, pH 8.5 at 20 °C with a protein: reservoir ratio of 1:4 and a total volume of 0.4 μl. The condition was already cryo-protected. Crystals were harvested by flash cooling in liquid nitrogen.

#### X-ray diffraction data collection

Native diffraction data were collected at Diamond Light Source (Harwell, UK) at beamline I03. Data were collected over 360° with 0.1° oscillation (Supplementary Table 1), integrated with DIALS^126^ and scaled/merged with Aimless^127^ from the CCP4 suite^128^. The crystals belonged to the space group P2_1_2_1_2, with unit cell dimensions of *a* = 101.23 Å, *b* = 31.5 Å, *c* = 59.5 Å and one molecule per asymmetric unit. The crystals diffracted up to 1.8 Å. BALBES was used to determine initial phases by Molecular Replacement against the entire PDB^129^. Manual building was done in COOT^130^ and refinement with REFMAC5. MOLPROBITY was used for model validation^131^. Statistics are listed in Supplementary Table 1. The coordinates and structure factors of the Odinarchaeota Vps25ΔN crystal structure were deposited in the Protein Data Bank under accession code 7PB9.

#### Chemical crosslinking of proteins

##### Vps22 dimer

Vps22 was diluted to 15 µM after the buffer exchange to XL-buffer (20 mM HEPES-NaOH (pH 7.5), 150 mM NaCl) and incubated with 16 mM EDC (Thermo Scientific) and 16 mM Sulfo-NHS (Thermo Fisher Scientific) or 2 mM BS3 [bis(sulfosuccinimidyl)suberate, Creativemolecules] on ice for 1 or 2 h, respectively. 55.6 mM Tris-HCl (pH 6.8) was added into the mixture to quench the crosslinking reaction. The sample was incubated on ice for 10 min to quench the crosslinking reactions. The samples were loaded in SDS-PAGE gels to separate individual or cross-linked proteins.

##### Ubiquitin and UEV

The full length (0.4 µM) or 1:130 amino acid residues containing UEV domain (5 µM) of Heimdall_14070 were incubated with 0, 1, 2, 4, 8, 16 and 24 µM Heimdall_14240 (wild-type or I45V) and 5 mM BS3 (Creativemolecules) on ice for 2 h. The crosslinking reaction was quenched by addition of 50 mM Tris-HCl (pH 6.8). The samples were loaded in SDS-PAGE gels to separate individual or cross-linked proteins. 50 mM Ammonium Bicarbonate (#A6141, Sigma) was used to dilute the sample using equal volume and reduced/alkylated using 10 mM Tris-|(2-carboxyethyl) phosphine hydrochloride (TCEP) (ACROS Organics)/40 mM 2-chromoacetamide ((CAA), Sigma) for 5 min at 70 °C. The samples were digested overnight at 37 °C with 1 µg trypsin (sequencing grade, Promega) per 100 µg of protein.

#### Chemical crosslinking coupled with mass spectrometric analysis

LC-MS was performed using Ultimate^®^ 3000 HPLC series for peptide concentration and separation. Nano Series ™ Standards Columns were then utilized to separate the samples. A linear gradient from 4–25% solvent B (0.1% formic acid in acetonitrile) was applied over 30 min, followed by 25–90% solvent B for 20 min. Peptides were eluted using at a rate of 250 nL min^−1^ using a Triversa Nanomate nano spray into the Orbitrap Fusion mass spectrometer (Thermo Scientific). Mass scan range of 375–1500 were used for the peptide precursors at 120 K resolution, with automatic gain control of 4 × 10^5^. Precursor ions range of 2–7 were isolated and fragmented using Higher-energy Collisional Dissociation (HCD) fragmentation using the Orbitrap detector at a resolution of 30 K. MS/MS fragmentation was performed using a collision energy of 33%, with a maximum injection time of 200 ms and automatic gain control of 1 × 10^4^. Dynamic exclusion duration of 45 s with 5 ppm tolerance was used for the selected precursor and its isotopes. The instrument was run with a cycle time of 2 s. 20 ul of the samples were injected into the nano LC-ESI-MS/MS using an Ultimate 3000/Orbitrap Fusion (Thermo Scientific) using a 60-min LC separation over a 50 cm column. The ProteoWizard MSConvert toolkit^132^ was used to convert the raw data files into.mgf format. Scaffold Proteome Software was used for sequence visualization and coverage. Cross-linked peptides were analysed using the Stavrox software^133^, using the in-built parameters for either BS3 or EDC. Precursor and fragment ion tolerance were set to 10 ppm. The spectra were manually inspected, and continuous fragment ions were expected to be seen for both peptides. Cross-linked peptides were identified in two replicate datasets. Detected peptides were listed in Supplementary Data 2.

### Reporting summary

Further information on research design is available in the Nature Research Reporting Summary linked to this article.

## Supplementary information

Supplementary Information

Reporting Summary

Peer Review File

Description of Additional Supplementary Files

Supplementary Data 1

Supplementary Data 2

## Data Availability

The crystallographic data generated in this study have been deposited in the PDB database under accession code 7PB9 [https://www.rcsb.org/structure/unreleased/7PB9]. Source data [for Fig. 2D, E, Fig. 3A, B, C and Supplementary Figs. 9, 10, 13, and 14] are provided with this paper.

## Source data

Source Data
