# Supplementary material for: Asgard archaea shed light on the evolutionary origins of the eukaryotic ubiquitin-ESCRT machinery
Source: Nat Commun. 2022 Jun 13;13:3398. doi: 10.1038/s41467-022-30656-2 (PMC9192718; doi:10.1038/s41467-022-30656-2)
Supplement: Supplementary file 1 — Supplementary Information [file 41467_2022_30656_MOESM1_ESM.pdf]

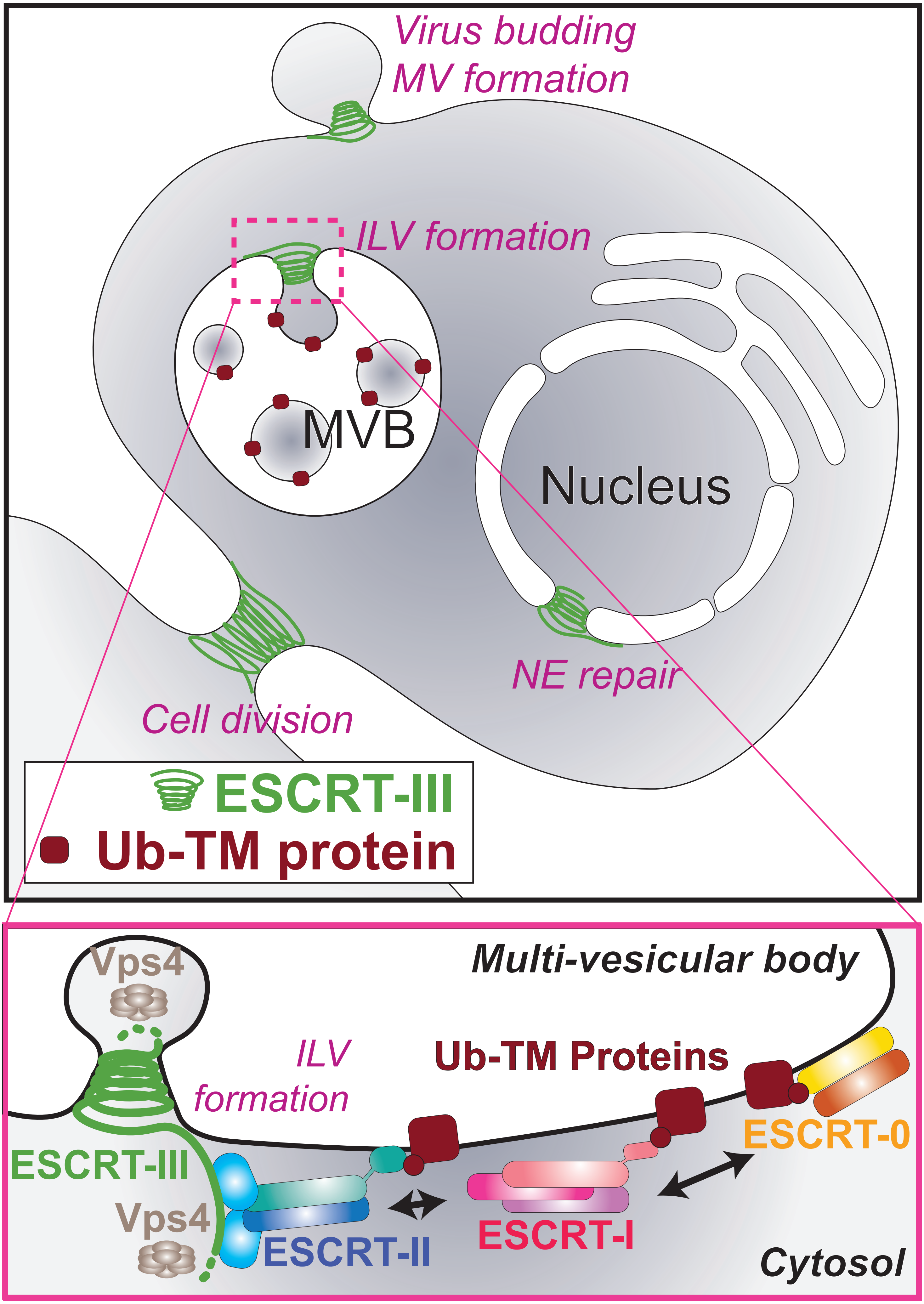

Virus budding
MV formation
ILV formation
MVB
Nucleus
NE repair
Cell division
ESCRT-III
Ub-TM protein
Multi-vesicular body
Vps4
ILV
formation
Ub-TM Proteins
ESCRT-III
Vps4
ESCRT-II
ESCRT-I
ESCRT-0
Cytosol

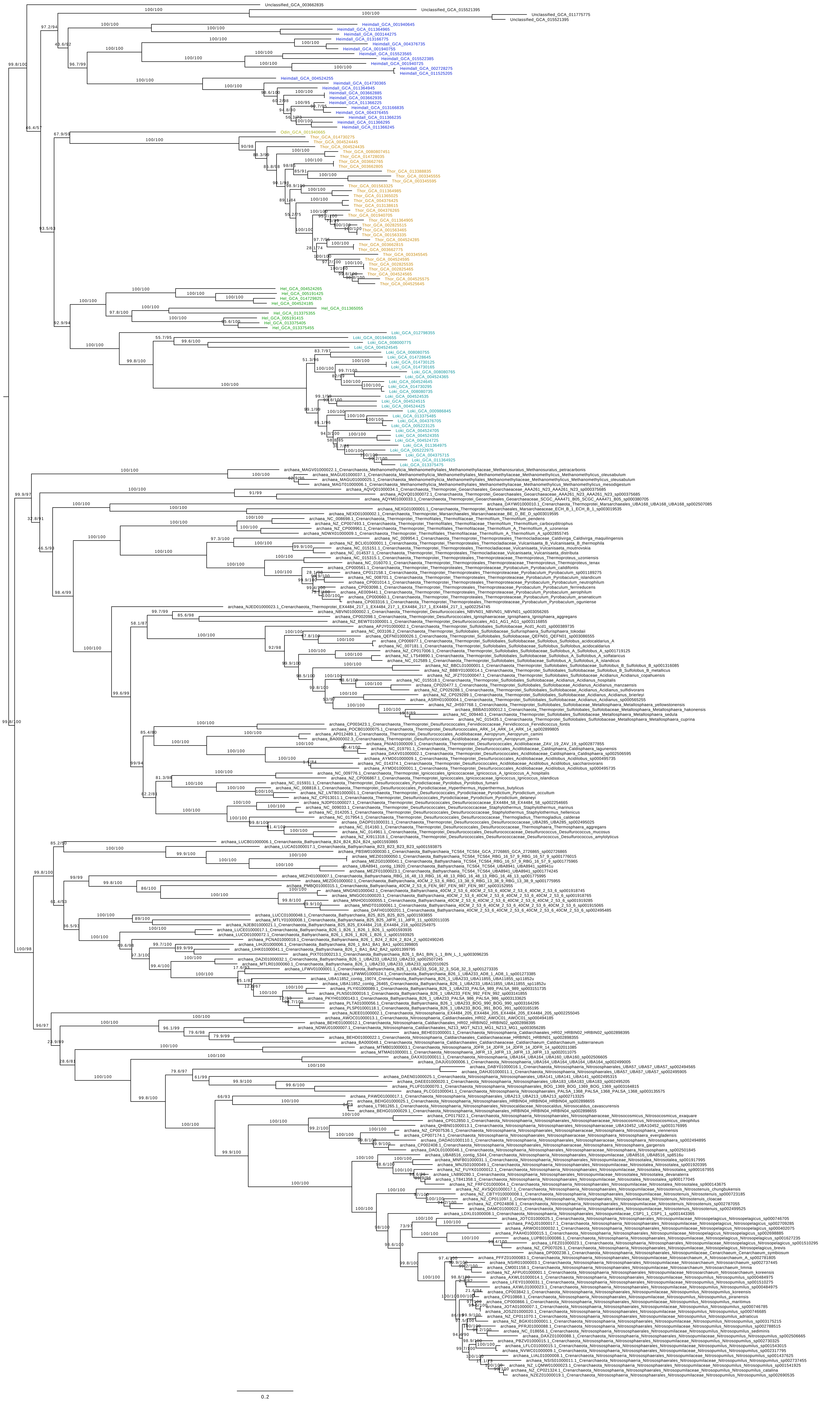
0.2

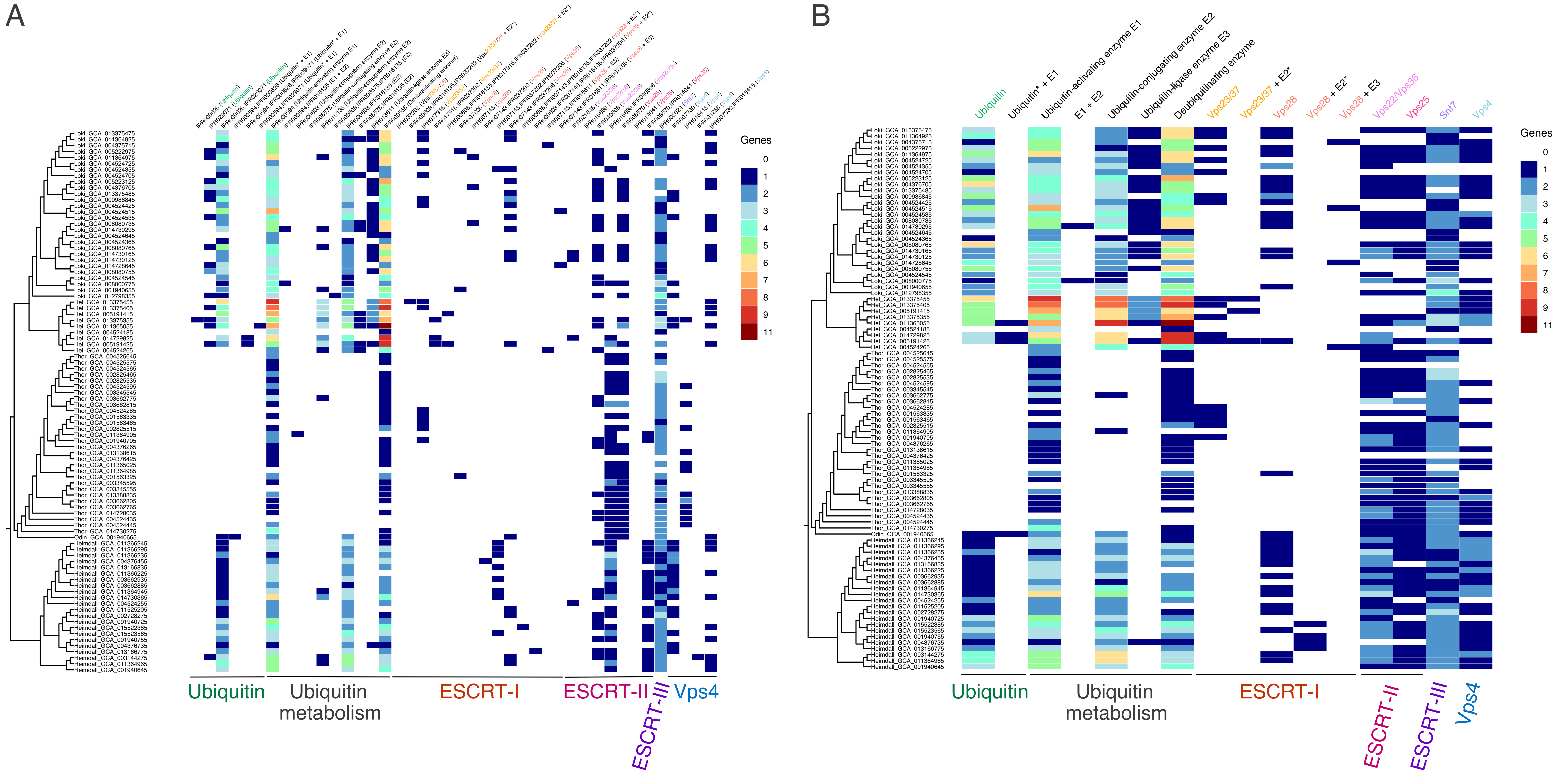
A
B
Genes
0
1
2
3
4
5
6
7
8
9
11
Ubiquitin
Ubiquitin* + E1
Ubiquitin-activating enzyme E1
E1 + E2
Ubiquitin-conjugating enzyme E2
Ubiquitin-ligase enzyme E3
Deubiquitinating enzyme
Vps23/37
Vps23/37 + E2*
Vps28
Vps28 + E2*
Vps28 + E3
Vps22/Vps36
Vps25
Snf7
Vps4
Ubiquitin
Ubiquitin metabolism
ESCRT-I
ESCRT-II
ESCRT-III
Vps4

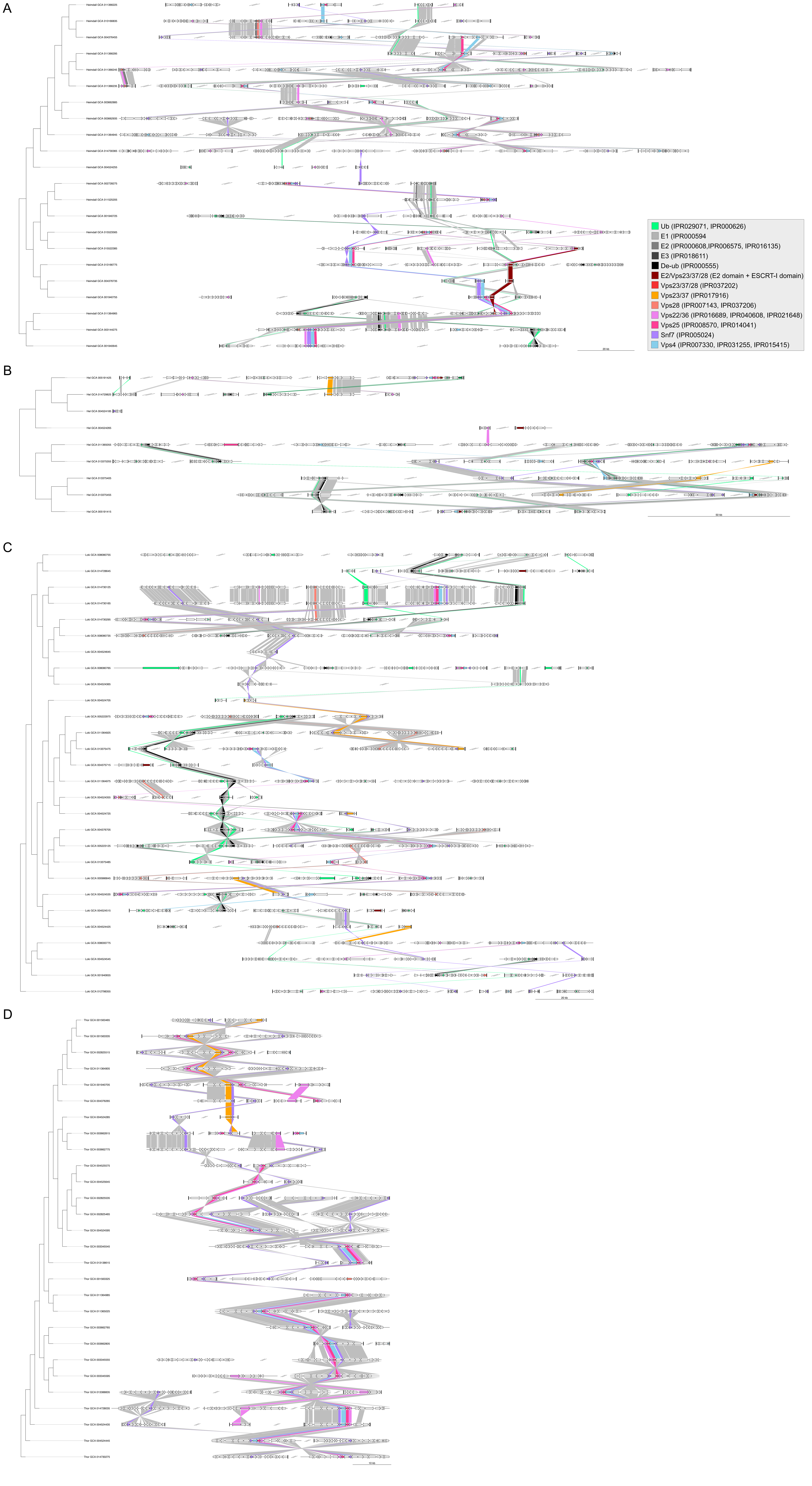
A
B
C
D
Ub (IPR029071, IPR000626)
E1 (IPR000594
E2 (IPR000608,IPR006575, IPR016135)
E3 (IPR018611)
De-ub (IPR000555)
E2/Vps23/37/28 (E2 domain + ESCRT-I domain)
Vps23/37/28 (IPR037202)
Vps23/37 (IPR017916)
Vps28 (IPR007143, IPR037206)
Vps22/36 (IPR016689, IPR040608, IPR021648)
Vps25 (IPR008570, IPR014041)
Snf7 (IPR005024)
Vps4 (IPR007330, IPR031255, IPR015415)

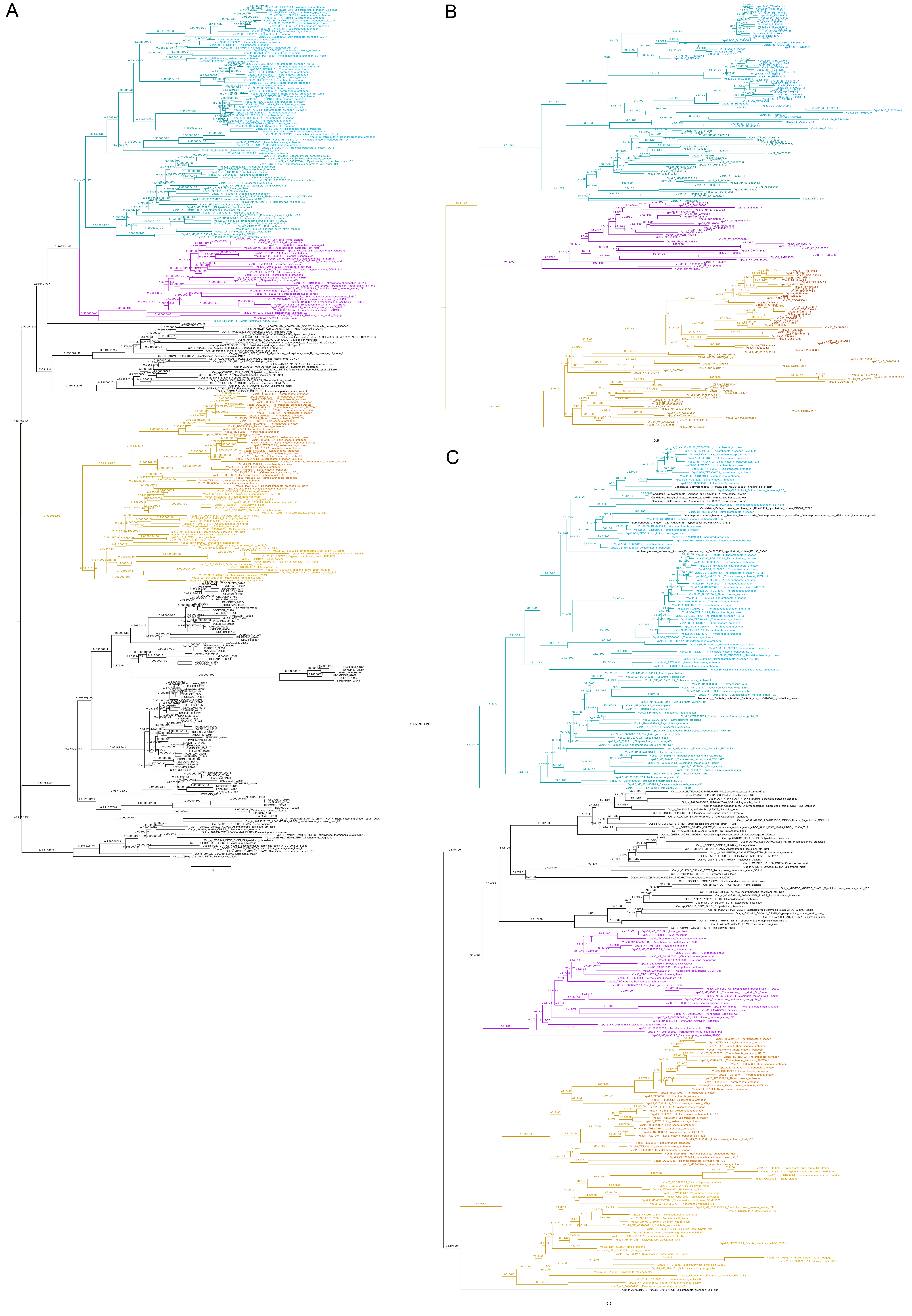
A
B
C
0.8
0.5
0.5

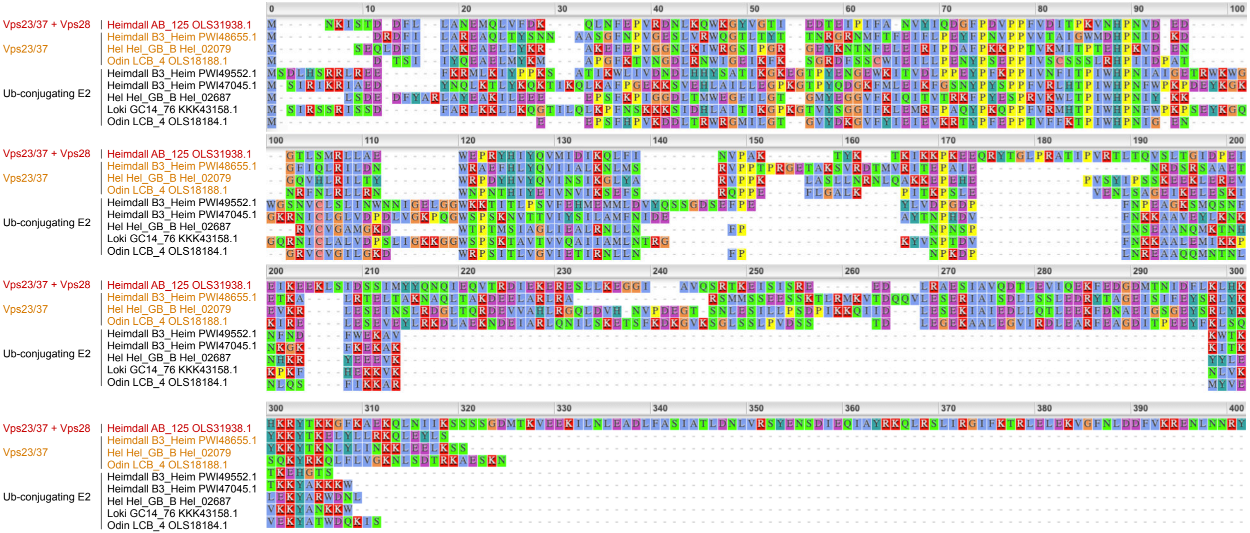

Vps23/37 + Vps28
Vps23/37
Ub-conjugating E2
Heimdall AB_125 OLS31938.1
Heimdall B3_Heim PWI48655.1
Hel Hel_GB_B Hel_02079
Odin LCB_4 OLS18188.1
Heimdall B3_Heim PWI49552.1
Heimdall B3_Heim PWI47045.1
Hel Hel_GB_B Hel_02687
Loki GC14_76 KKK43158.1
Odin LCB_4 OLS18184.1

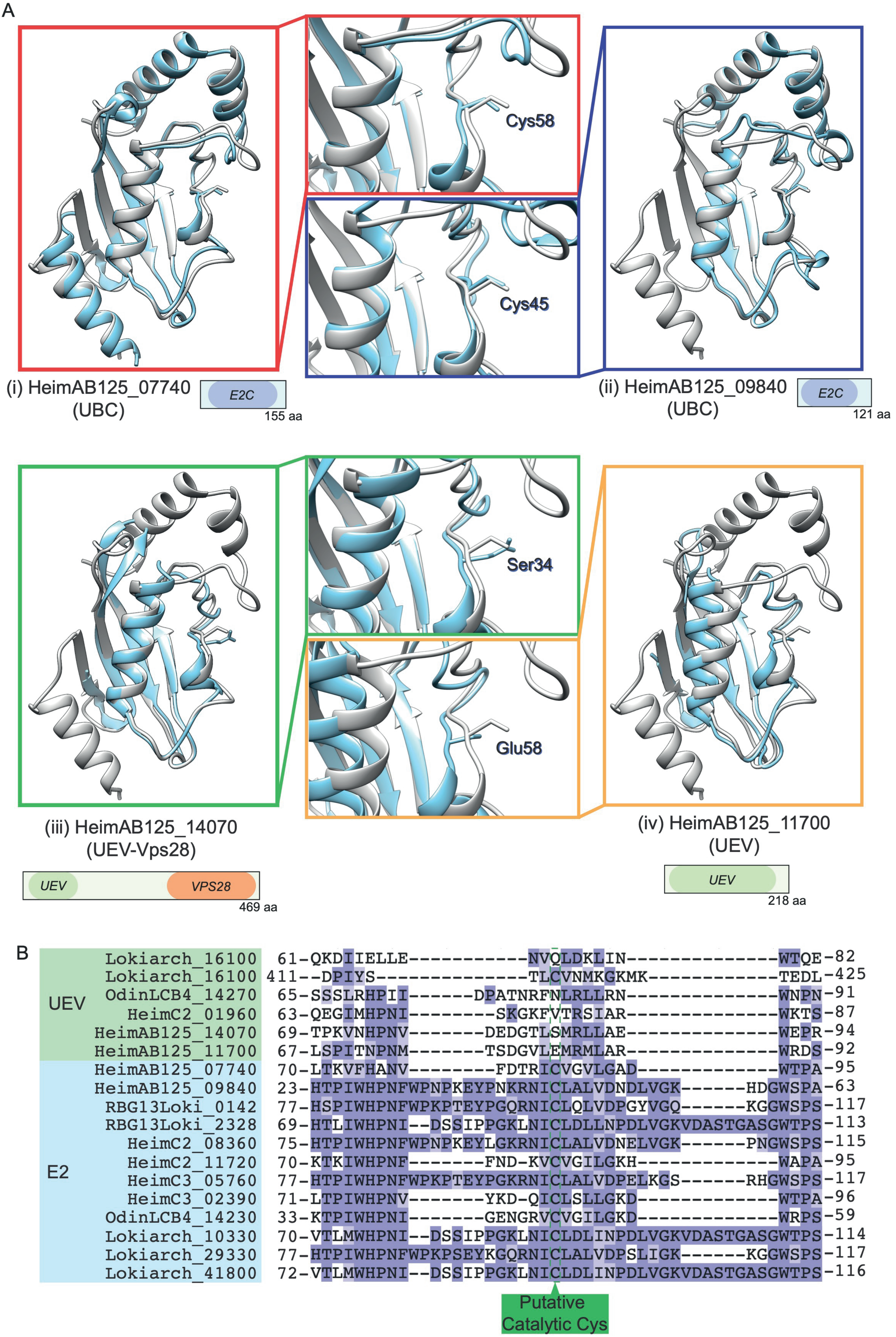
A
Cys58
Cys45
(i) HeimAB125_07740
(UBC)
E2C
155 aa
(ii) HeimAB125_09840
(UBC)
E2C
121 aa
Ser34
Glu58
(iii) HeimAB125_14070
(UEV-Vps28)
UEV
VPS28
469 aa
(iv) HeimAB125_11700
(UEV)
UEV
218 aa
B
UEV
E2
Lokiarch_16100 61-QKDIIELLE-----------NVQLDKLIN--------------WTQE-82
Lokiarch_16100 411--DPIYS---------------TLCVNMKGKMK-------------TEDL-425
OdinLCB4_14270 65-SSSLRHPII------DPATNRFNLRLLRN--------------WNPN-91
HeimC2_01960 63-QEGIMHPNI--------SKGKFVTRSIAR--------------WKTS-87
HeimAB125_14070 69-TPKVNHPNV-------DEDGTLSMRLLAE--------------WEPR-94
HeimAB125_11700 67-LSPITNPNM-------TSDGVLEMRMLAR--------------WRDS-92
HeimAB125_07740 70-LTKVFHANV--------FDTRICVGVLGAD-------------WTPA-95
HeimAB125_09840 23-HTPIWHPNFWPNPKEYPNKRNICLALVDNDLVGK------HDGWSPA-63
RBG13Loki_0142 77-HSPIWHPNFWPKPTEYPGQRNICLQLVDPGYVGQ------KGGWSPS-117
RBG13Loki_2328 69-HTLIWHPNI--DSSIPPGKLNICLDLLNPDLVGKVDASTGASGWTPS-113
HeimC2_08360 75-HTPIWHPNFWPNPKEYLGKRNICLALVDNELVGK------PNGWSPS-115
HeimC2_11720 70-KTKIWHPNF-------FND-KVCVGILGKH--------------WAPA-95
HeimC3_05760 77-HTPIWHPNFWPKPTEYPGKRNICLALVDPELKGS------RHGWSPS-117
HeimC3_02390 71-LTPIWHPNV-------YKD-QICLSLLGKD-------------WTPA-96
OdinLCB4_14230 33-KTPIWHPNI-------GENGRVCVGILGKD-------------WRPS-59
Lokiarch_10330 70-VTLMWHPNI--DSSIPPGKLNICLDLINPDLVGKVDASTGASGWTPS-114
Lokiarch_29330 77-HTPIWHPNFWPKPSEYKGQRNICLALVDPSLIGK------KGGWSPS-117
Lokiarch_41800 72-VTLMWHPNI--DSSIPPGKLNICLDLINPDLVGKVDASTGASGWTPS-116
Putative Catalytic Cys

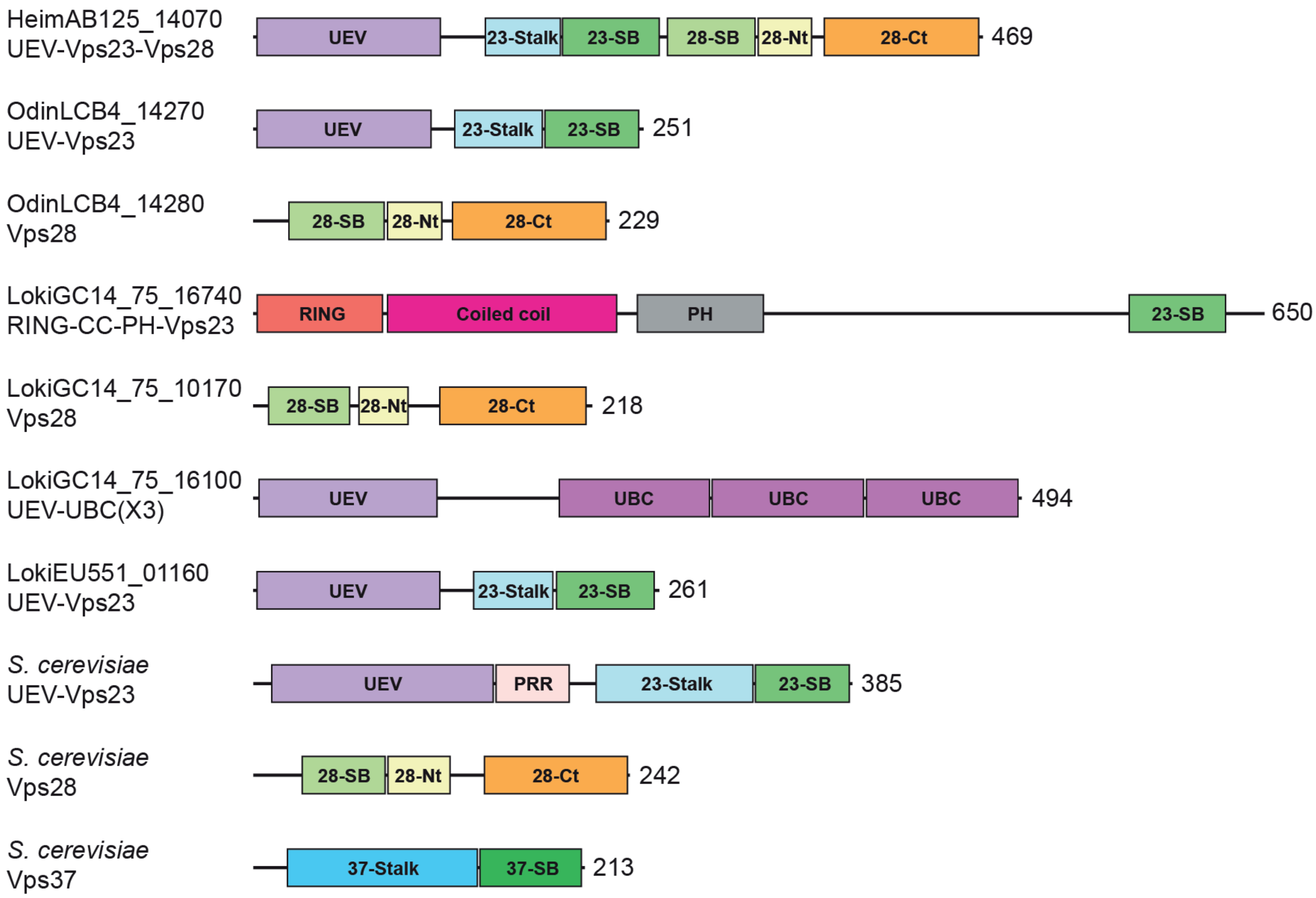
HeimAB125_14070
UEV-Vps23-Vps28
UEV
23-Stalk
23-SB
28-SB
28-Nt
28-Ct
469
OdinLCB4_14270
UEV-Vps23
UEV
23-Stalk
23-SB
251
OdinLCB4_14280
Vps28
28-SB
28-Nt
28-Ct
229
LokiGC14_75_16740
RING-CC-PH-Vps23
RING
Coiled coil
PH
23-SB
650
LokiGC14_75_10170
Vps28
28-SB
28-Nt
28-Ct
218
LokiGC14_75_16100
UEV-UBC(X3)
UEV
UBC
UBC
UBC
494
LokiEU551_01160
UEV-Vps23
UEV
23-Stalk
23-SB
261
S. cerevisiae
UEV-Vps23
UEV
PRR
23-Stalk
23-SB
385
S. cerevisiae
Vps28
28-SB
28-Nt
28-Ct
242
S. cerevisiae
Vps37
37-Stalk
37-SB
213

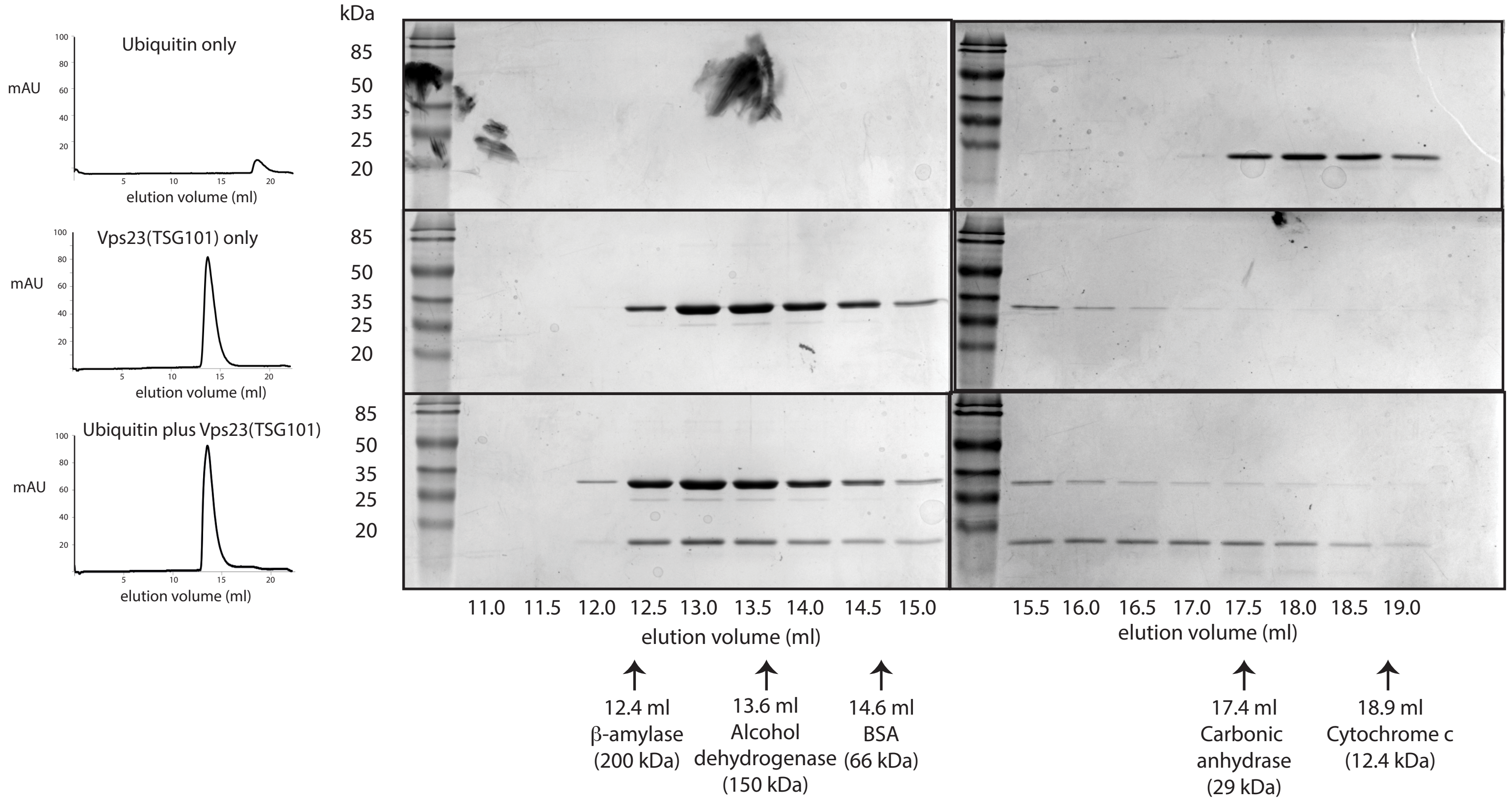

Ubiquitin only
mAU
elution volume (ml)
Vps23(TSG101) only
mAU
elution volume (ml)
Ubiquitin plus Vps23(TSG101)
mAU
elution volume (ml)
kDa
85
50
35
25
20
11.0 11.5 12.0 12.5 13.0 13.5 14.0 14.5 15.0
elution volume (ml)
15.5 16.0 16.5 17.0 17.5 18.0 18.5 19.0
elution volume (ml)
12.4 ml β-amylase (200 kDa)
13.6 ml Alcohol dehydrogenase (150 kDa)
14.6 ml BSA (66 kDa)
17.4 ml Carbonic anhydrase (29 kDa)
18.9 ml Cytochrome c (12.4 kDa)

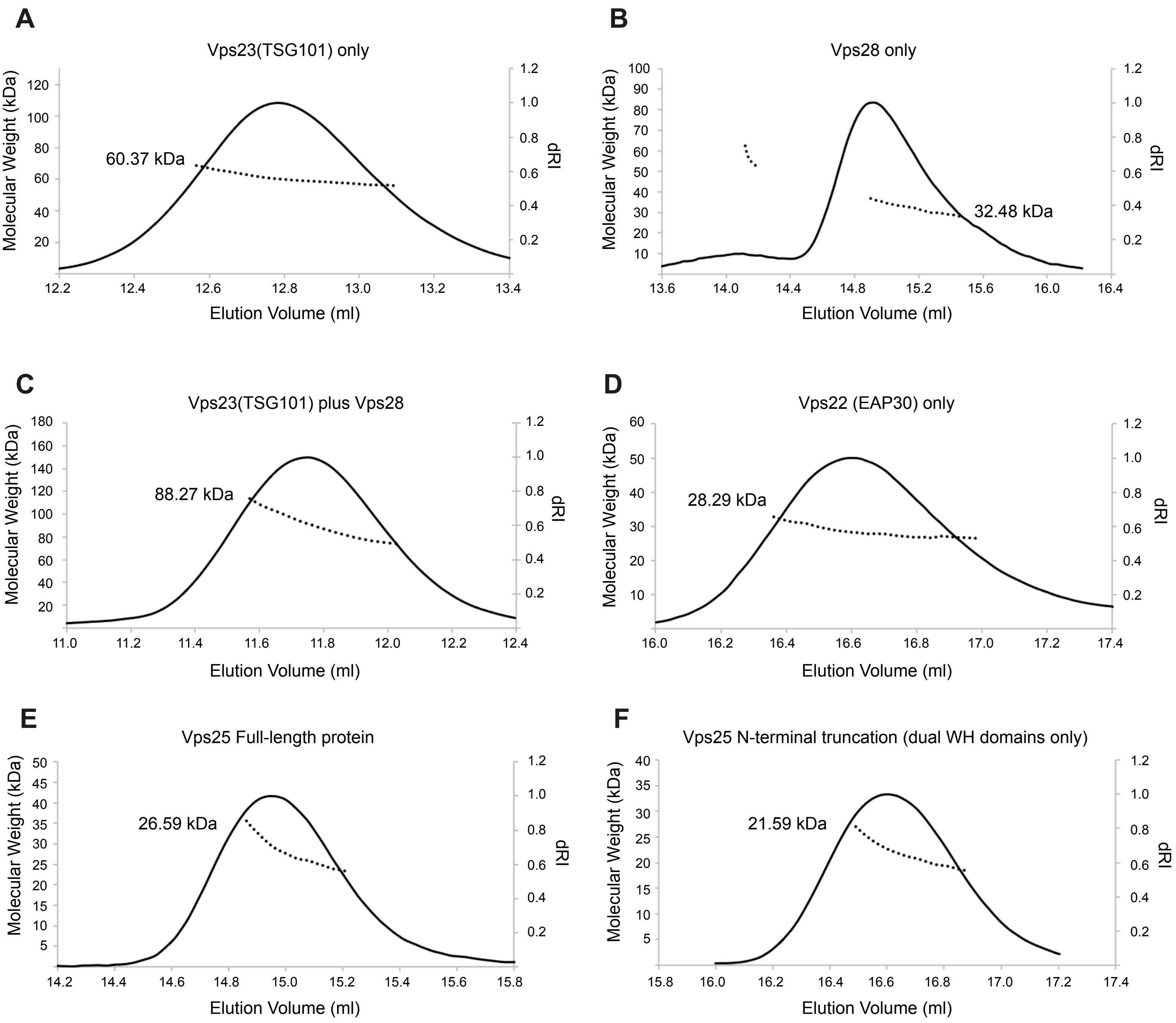
A
Vps23(TSG101) only
60.37 kDa
Molecular Weight (kDa)
dRI
Elution Volume (ml)
B
Vps28 only
32.48 kDa
Molecular Weight (kDa)
dRI
Elution Volume (ml)
C
Vps23(TSG101) plus Vps28
88.27 kDa
Molecular Weight (kDa)
dRI
Elution Volume (ml)
D
Vps22 (EAP30) only
28.29 kDa
Molecular Weight (kDa)
dRI
Elution Volume (ml)
E
Vps25 Full-length protein
26.59 kDa
Molecular Weight (kDa)
dRI
Elution Volume (ml)
F
Vps25 N-terminal truncation (dual WH domains only)
21.59 kDa
Molecular Weight (kDa)
dRI
Elution Volume (ml)

A

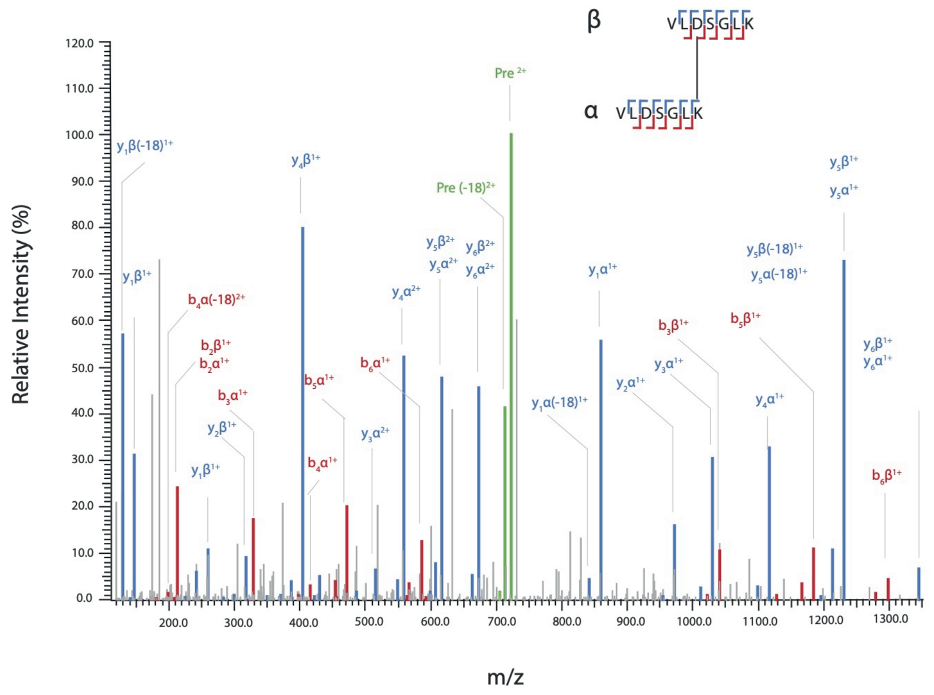
β VLDSGLK
α VLDSGLK
Relative Intensity (%)
m/z
Pre $^{2+}$
Pre (-18)$^{2+}$
$y_1\beta(-18)^{1+}$
$y_1\beta^{1+}$
$y_4\beta^{1+}$
$b_4\alpha(-18)^{2+}$
$b_2\beta^{1+}$
$b_2\alpha^{1+}$
$b_3\alpha^{1+}$
$y_2\beta^{1+}$
$y_1\beta^{1+}$
$b_5\alpha^{1+}$
$b_4\alpha^{1+}$
$y_3\alpha^{2+}$
$y_4\alpha^{2+}$
$y_5\beta^{2+}$
$y_5\alpha^{2+}$
$y_6\beta^{2+}$
$y_6\alpha^{2+}$
$b_6\alpha^{1+}$
$y_1\alpha(-18)^{1+}$
$y_1\alpha^{1+}$
$y_2\alpha^{1+}$
$y_3\alpha^{1+}$
$b_3\beta^{1+}$
$b_5\beta^{1+}$
$y_4\alpha^{1+}$
$y_5\beta(-18)^{1+}$
$y_5\alpha(-18)^{1+}$
$y_5\beta^{1+}$
$y_5\alpha^{1+}$
$y_6\beta^{1+}$
$y_6\alpha^{1+}$
$b_6\beta^{1+}$

B

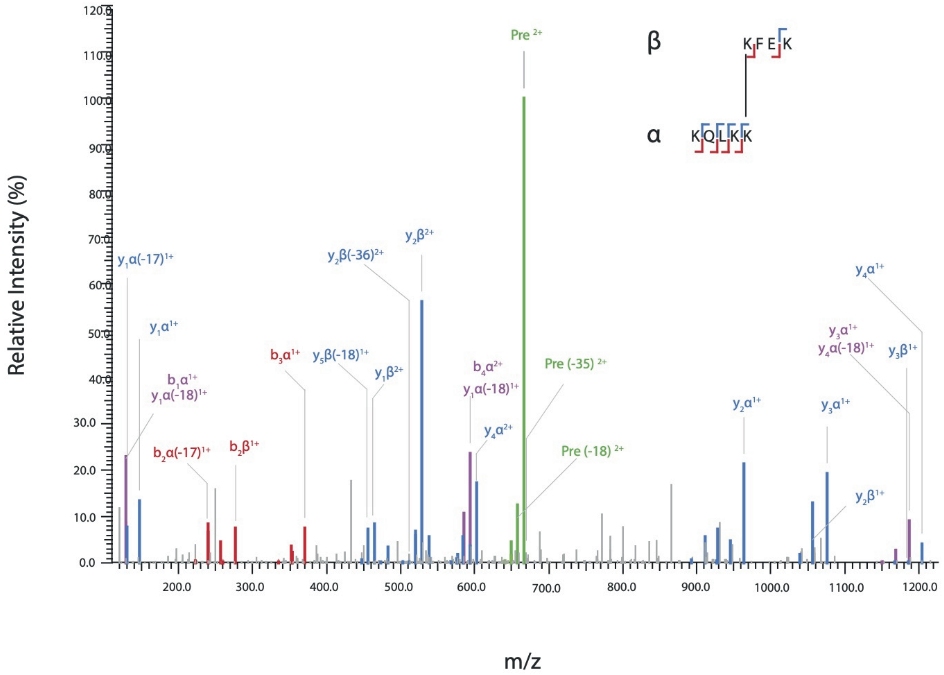
β KFEK
α KQLKK
Relative Intensity (%)
m/z
Pre $^{2+}$
Pre (-35) $^{2+}$
Pre (-18) $^{2+}$
$y_1\alpha(-17)^{1+}$
$y_1\alpha^{1+}$
$b_1\alpha^{1+}$
$y_1\alpha(-18)^{1+}$
$b_2\alpha(-17)^{1+}$
$b_2\beta^{1+}$
$b_3\alpha^{1+}$
$y_2\beta(-18)^{1+}$
$y_1\beta^{2+}$
$y_2\beta(-36)^{2+}$
$y_2\beta^{2+}$
$b_4\alpha^{2+}$
$y_1\alpha(-18)^{1+}$
$y_4\alpha^{2+}$
$y_2\alpha^{1+}$
$y_3\alpha^{1+}$
$y_3\alpha^{1+}$
$y_4\alpha(-18)^{1+}$
$y_3\beta^{1+}$
$y_4\alpha^{1+}$
$y_2\beta^{1+}$

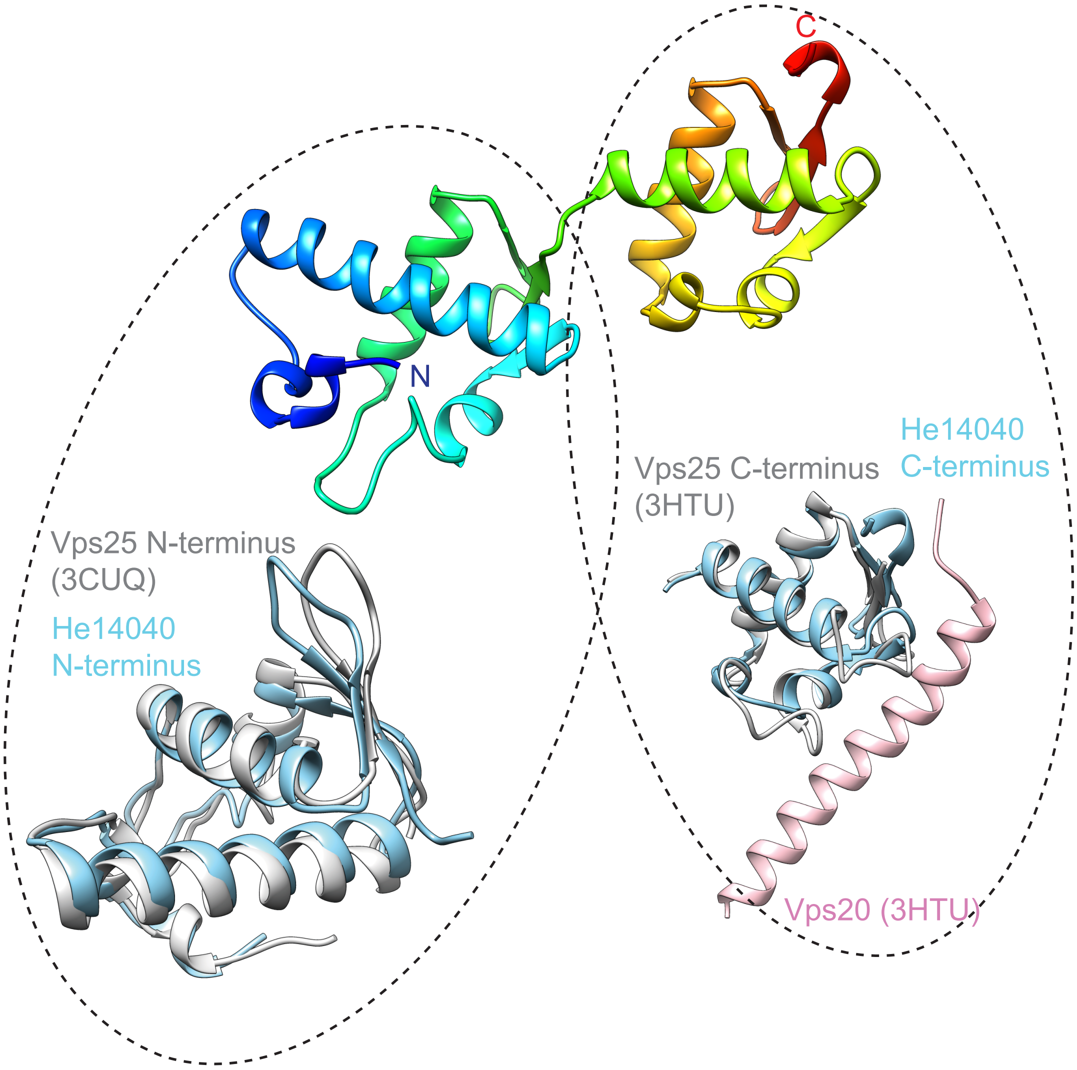
C
N
Vps25 N-terminus
(3CUQ)
He14040
N-terminus
Vps25 C-terminus
(3HTU)
He14040
C-terminus
Vps20 (3HTU)

A

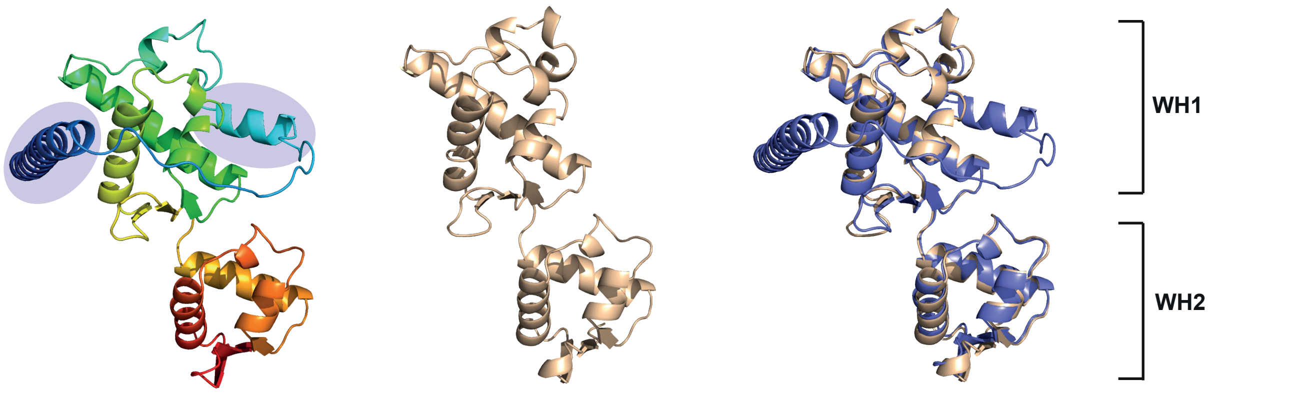

AF2 model Odinarchaeota Vps25 ESCRT-II (blue to red N- to C- terminus)

Odinarchaeota Vps25 crystal structure (PDB: 7PB9)

Superposition of the Odinarchaeota AF2 Vps25 model (slate) with 7PB9 (wheat)

B

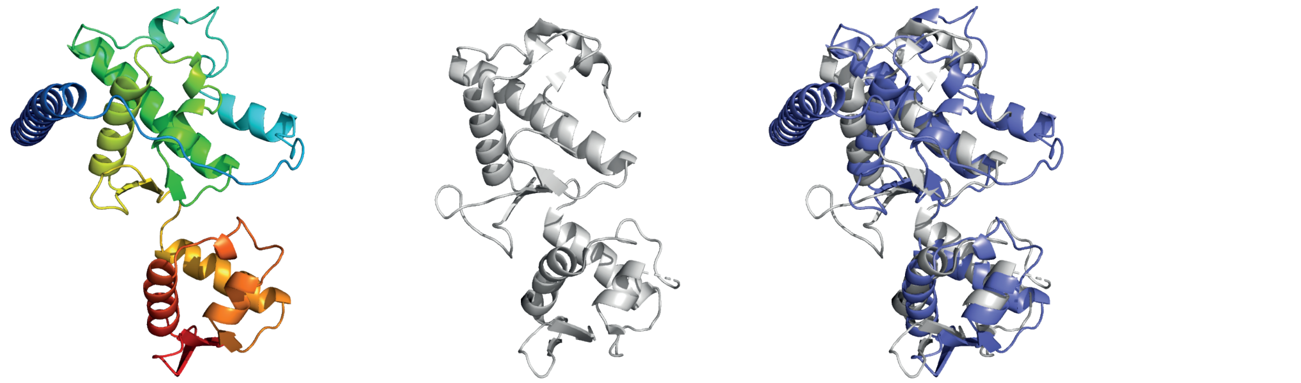

AF2 model Odinarchaeota Vps25 ESCRT-II (blue to red N- to C- terminus)

Yeast Vps25 crystal structure (PDB: 1XB4)

Superposition of the Odinarchaeota AF2 Vps25 model (slate) with 1XB4 (grey)

C

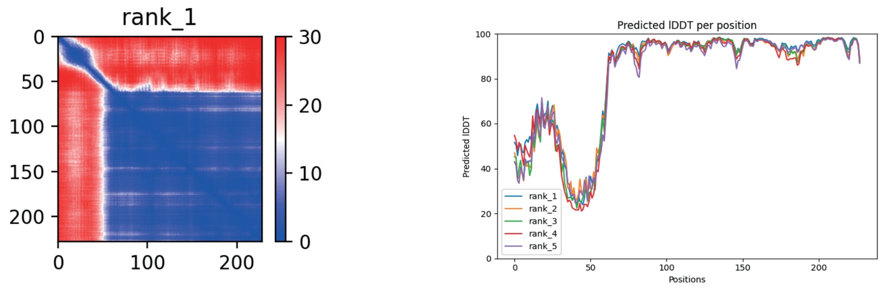

D

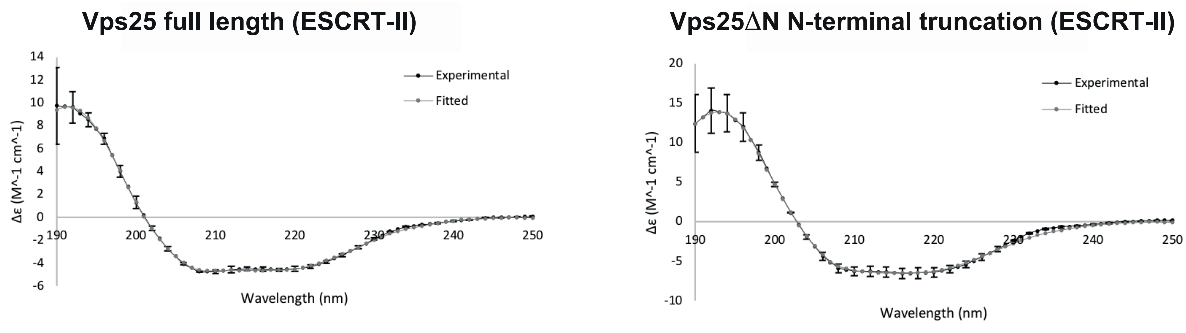

	α-helix	β-sheet	other
Vps25ΔN (CD)	52.1%	17.8%	30.1%
Vps25ΔN (crystal struc)	54.3%	12.7%	33.0%
Vps25 (CD)	56.6%	15.1%	28.3%
Vps25 (AF2 model)	57.0%	9.6%	33.3%

E

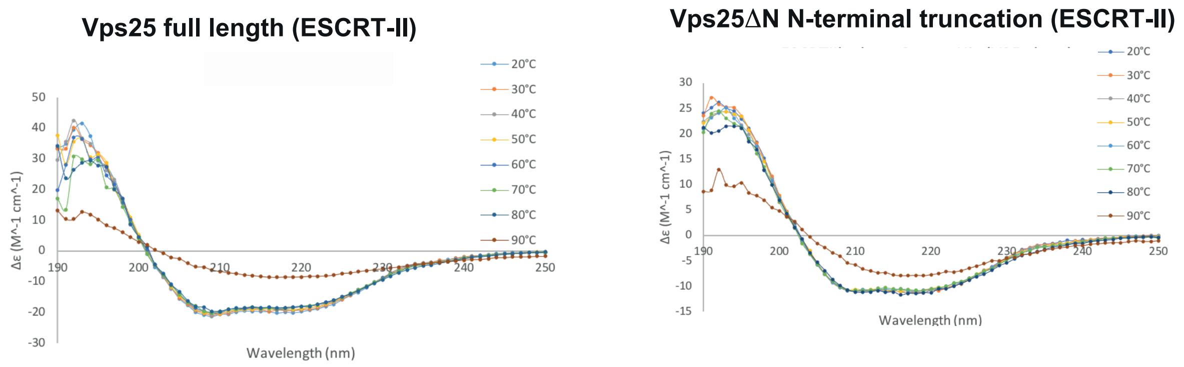

A

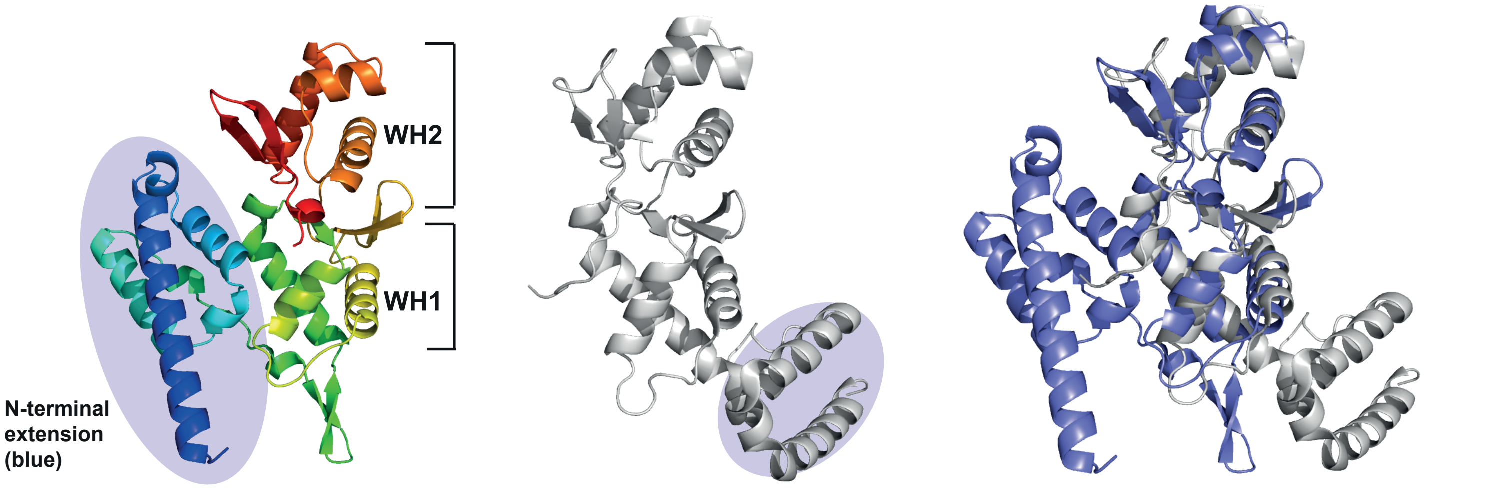

AF2 model Odinarchaeota Vps22 ESCRT-II (blue to red N- to C- terminus)

Human Vps22 crystal structure (PDB: 3CUQ)

Superposition of the Odinarchaeota AF2 Vps22 model (slate) with the human Vps22 crystal structure (grey)

B

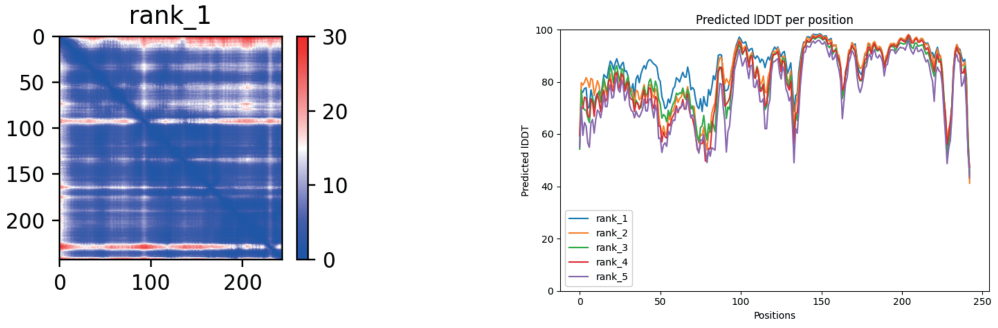

C

Vps22 (ESCRT-II EAP30 domain)

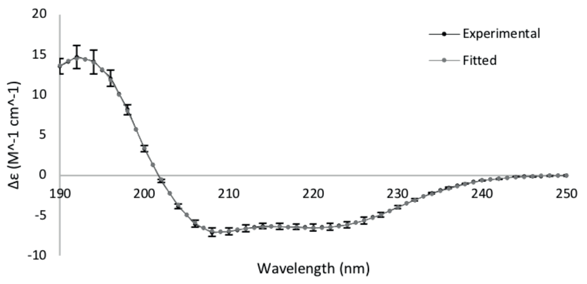

	α-helix	β-strand	β-sheet	other
**Vps22 (AF2 model)**	**59.6%**	**14.4%**(as β-turn)	**0%**	**25.9%**
**Vps22 (CD measured)**	**59.4%**	all β-turn measured as 'other'	**0.8%**	**39.8%**

D

Vps22 (ESCRT-II EAP30 domain)

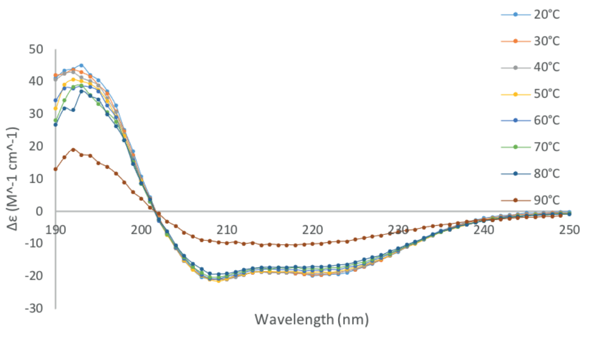

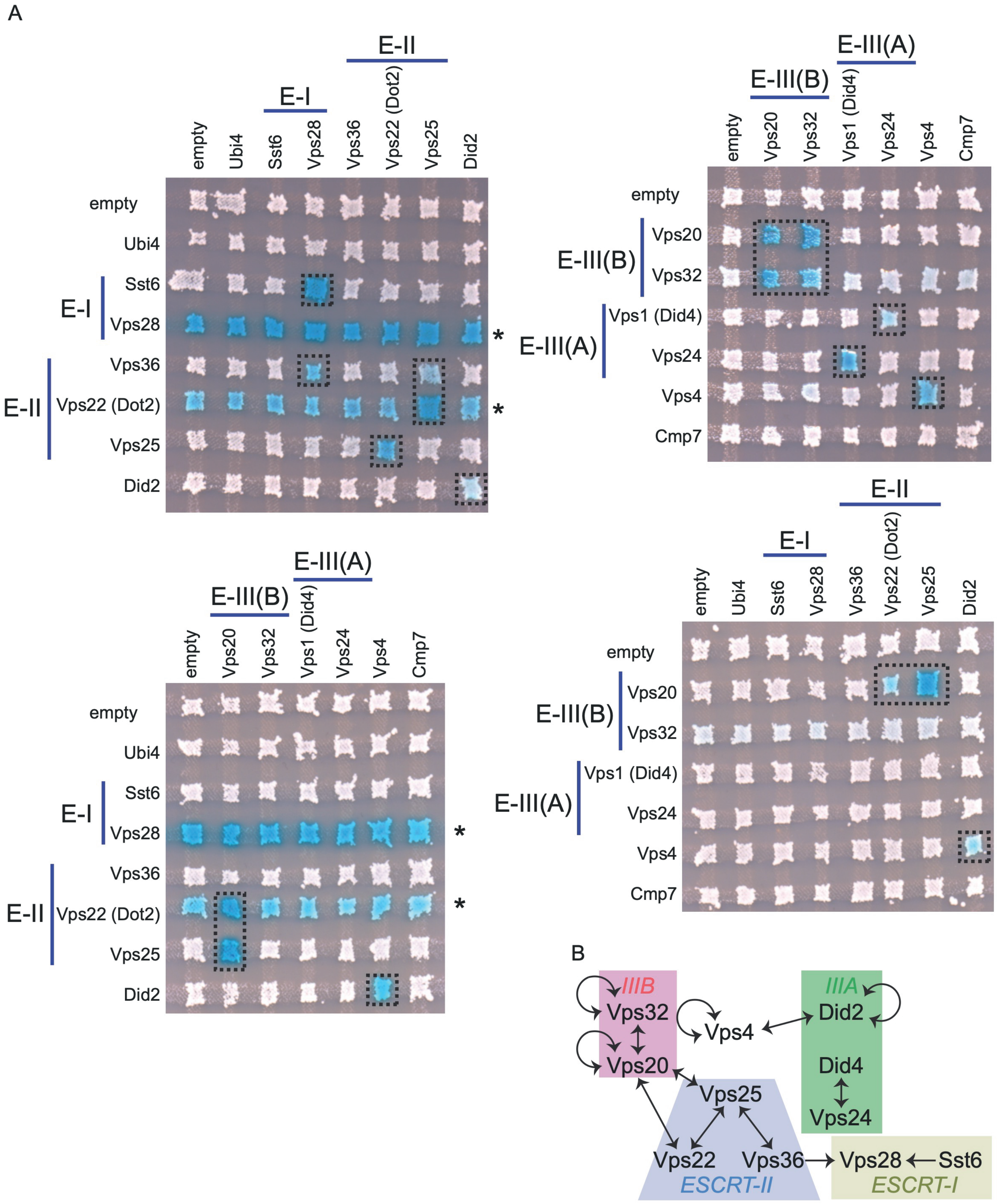

A
E-I
E-II
empty
Ubi4
Sst6
Vps28
Vps36
Vps22 (Dot2)
Vps25
Did2
*
E-III(A)
E-III(B)
Vps20
Vps32
Vps1 (Did4)
Vps24
Vps4
Cmp7
B
IIIB
IIIA
Did2
Did4
ESCRT-II
ESCRT-I

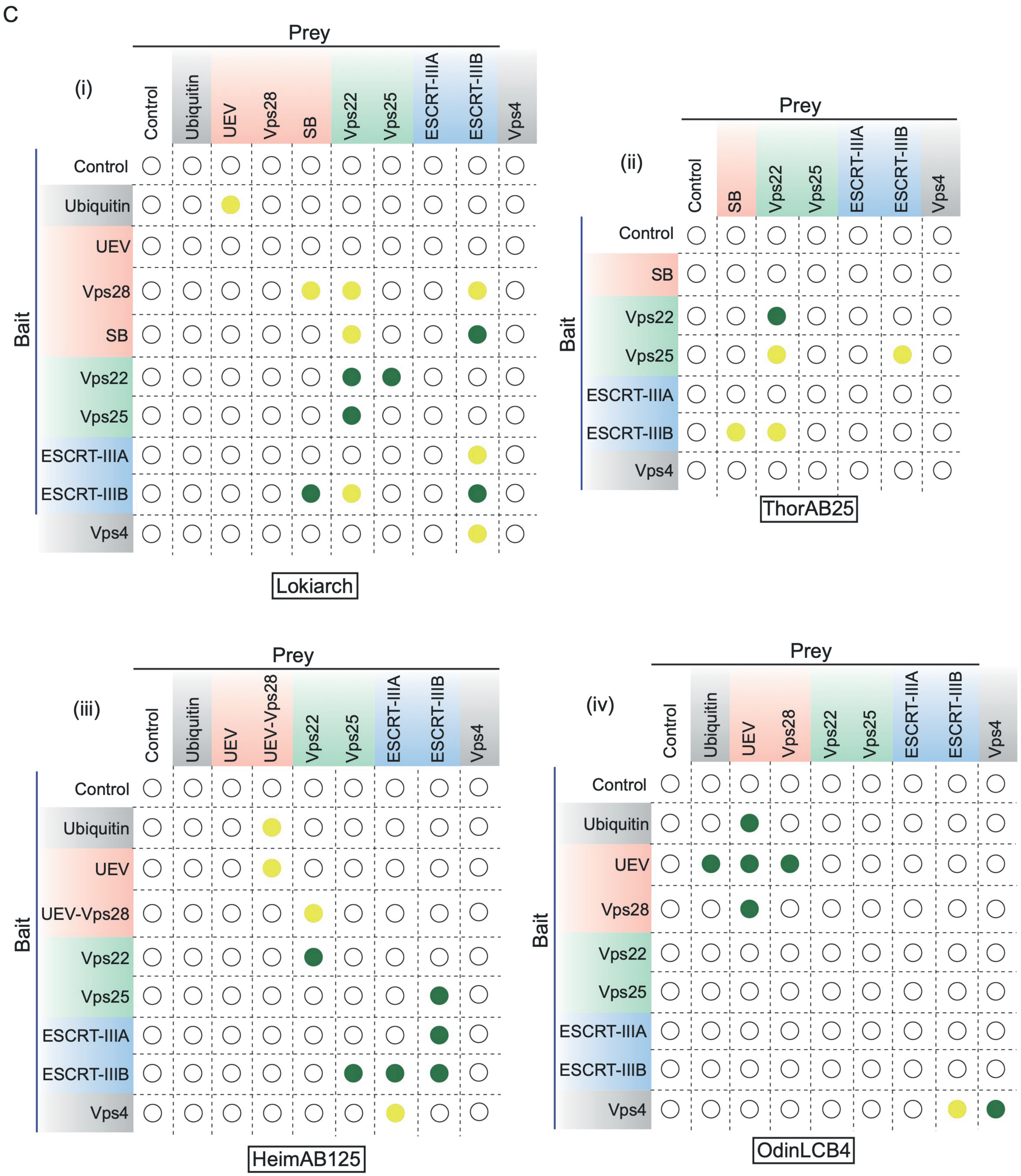
C
(i)
Prey
Control
Ubiquitin
UEV
Vps28
SB
Vps22
Vps25
ESCRT-IIIA
ESCRT-IIIB
Vps4
Bait
Lokiarch
(ii)
Prey
Control
SB
Vps22
Vps25
ESCRT-IIIA
ESCRT-IIIB
Vps4
Bait
ThorAB25
(iii)
Prey
Control
Ubiquitin
UEV
UEV-Vps28
Vps22
Vps25
ESCRT-IIIA
ESCRT-IIIB
Vps4
Bait
HeimAB125
(iv)
Prey
Control
Ubiquitin
UEV
Vps28
Vps22
Vps25
ESCRT-IIIA
ESCRT-IIIB
Vps4
Bait
OdinLCB4

Supplemental Figure Legends

Supplementary Figure 1: Schematic representation of the Eukaryotic ESCRT pathway. Top panel. Some physiological processes regulated by ESCRT machinery. Bottom panel. Schematic representation of Ubiquitin-ESCRT molecular mechanisms leading to membrane reorganization. Ubiquitin is recognised by the UEV domains of Vps23 (and some Vps37 homologues) of the Vps37/Vps23/Vps28 ESCRT-I ternary complex. The C-terminal alpha-helical bundle of Vps28 interacts with the GLUE/zinc finger domain of ESCRT-II to bridge the ESCRT-I and -II subcomplexes. The eukaryotic ESCRT-II complex in yeast and human systems forms a Y-shaped tetramer consisting of a Vps22/Vps36 stalk leading to a splayed Vps25 dimer. The core of all the related ESCRT-II subunits consist of tandem WH domains. The splayed Vps25 dimer then recruits the ESCRT-III membrane scission machinery with a geometry that facilitates the formation of spiral ESCRT-III filaments.

Supplementary Figure 2. Phylogenetic classification of Asgard archaeal bins into robust lineages using 15 co-locating ribosomal proteins. Genomes downloaded from public databases were reannotated and searched for ribosomal proteins from a set of 15 encoded in the same gene cluster. Sequences of 5 or more of these ribosomal proteins encoded in the same contig were aligned, trimmed, and concatenated for phylogenetic reconstruction. A maximum-likelihood phylogeny was reconstructed using IQTree under the LG+C60+R4+F model. Support values represent approximate likelihood ratio tests (left) and ultrafast bootstrap (right) based on 1000 pseudoreplicates each. Outgroup sequences were all non-Korarchaeal Crenarchaeota species representatives available at GTDB on December 5th. Only genomes confidently classified as Heimdall-, Hel-, Loki-, Odin- and Thorarchaea (colored) were used for further analyses. This phylogenomic classification guided the use of MAG data for bioinformatic analyses in Figure 1, Supplementary Figure 3 and Supplementary Figure 5.

Supplementary Figure 3. ESCRT/Ub domain detection. Number of genes with protein domains related to ESCRT and Ubiquitin metabolism in Asgard archaea. (A) Number of genes containing specific Interpro domains and domain combinations. Parentheses include the gene families that the corresponding domains represent. (B) Number of genes containing their corresponding Interpro domains or domain combinations shown in (A). Asterisks behind 'Ubiquitin' and 'E2' indicate genes with domains that are characteristic of these proteins, but that, in combination with E1 and ESCRT-I domains (respectively), are likely to simply represent the latter. IPR029071 = 'Ubiquitin-like domain superfamily', IPR000626 = 'Ubiquitin-like domain', IPR000594 = 'THIF-type NAD/FAD binding fold', IPR000608 = 'Ubiquitin-conjugating enzyme E2', IPR006575 = 'RWD domain', IPR016135 = 'Ubiquitin-conjugating enzyme/RWD-like', IPR018611 = 'E3 UFM1-protein ligase 1', IPR000555 = 'JAB1/MPN/MOV34 metalloenzyme domain', IPR017916 = 'Steadiness box (SB) domain', IPR007143 = 'Vacuolar protein sorting-associated Vps28', IPR037206 = 'VPS28, C-terminal domain superfamily', IPR037202 = 'ESCRT assembly domain', IPR016689 = 'ESCRT-2 complex, Snf8', IPR040608 = 'Snf8/Vps36 family', IPR021648 = 'Vacuolar protein sorting protein 36, GLUE domain', IPR008570 = 'ESCRT-II complex, Vps25 subunit', IPR014041 = 'ESCRT-II complex, Vps25 subunit, N-terminal winged helix', IPR005024 = 'Snf7 family', IPR007330 = 'MIT domain', IPR031255 = 'Vacuolar protein sorting-associated protein 4', IPR015415 = 'Vps4 oligomerisation, C-terminal'. The presence/ absence results obtained by this analysis serve as a basis for the results shown in Fig. 1, Fig S3-S5

Supplementary Figure 4. Synteny plot of ESCRT-Ub genes in Asgard archaea. Maps around genes containing ESCRT and Ubiquitin domains for Heimdall- (A), Thor- (B), Hel- (C) and Lokiarchaeota (D), using GenoPlotR. Arrows represent genes and are colored if their products were annotated as containing diagnostic domains for Ub/ESCRT proteins (see legend in A). Genome regions are plotted at 10 kb of ubiquitin or ESCRT protein-encoding genes (colored), or until a contig boundary (thicker vertical lines). Similarity lines indicate best-reciprocal BLAST-p hits with an e-value lower than 1e-5. This depiction of the gene neighbourhoods of ESCRT/Ub genes complements the co-localisation analysis in Fig. 1B and expands on the highlighted reference taxa in Fig. 1C

Supplementary Figure 5. Phylogenetic reconstruction of ESCRT-II genes. (A) Unrooted maximum likelihood phylogenetic tree of Vps22 (blue), Vps36 (purple), Vps25 (orange) and outgroup (black) sequences corresponding to the same dataset as Fig 1D. The tree was reconstructed using IQ-Tree under the LG+C60+R4+F+PMSF model. Support values represent transfer-bootstrap expectation (left) values or standard Felsenstein bootstrap proportions (right) based on 100 bootstrap pseudorreplicates. This phylogeny is the fully labeled result behind the collapsed tree in Fig 1D. (B) Unrooted maximum likelihood phylogenetic tree of the same sequence dataset as (A) without outgroup sequences. The tree was reconstructed using IQ-Tree under the LG+C60+R4+F model. Support values represent approximate likelihood ratio tests (left) and ultrafast bootstrap (right) based on 1000 pseudoreplicates each. This phylogeny shows that the obtained results are robust to the inclusion of distant outgroup sequences. (C) Unrooted maximum likelihood phylogenetic tree of the same sequence dataset as (B) plus three small outgroup clades included in (A) (black), and additional homologs identified through a Psiblast search against NR (see Methods) and classified as neither eukaryotic or Asgard archaeal (black). The tree was reconstructed using IQ-Tree under the LG+C20+R4+F model. Support values represent approximate likelihood ratio tests (left) and ultrafast bootstrap (right) based on 1000 pseudoreplicates each. This phylogeny indicates that no phylogenetically informative homologs outside of Asgard archaea or eukaryotes were missed in the trees shown in Fig. 1D, Fig S5A and Fig S5B.

Supplementary Figure 6. Alignment of amino acid sequences of Asgard E2- and UEV-domain-containing proteins reveals domain architecture differences. Alignment between genes identified as Ubiquitin-conjugating enzyme E2 (black), UEV-containing Vps23/Vps37 (orange) or as a gene fusion between Vps23/Vps37 and Vps28 (red), performed with Mafft E-INS-i (v7.450) and visualized with http://was.bi/. For visualization purposes, the alignment is only shown until position 400 (OLS31938.1 continues for 138 more residues).

Supplementary Figure 7. A. Structural modelling identifies ubiquitin-binding enzyme (E2) and potential UEV domain proteins in Heimdallarchaeota. The model structure of the E2-like domain of Heimdallarchaeota (light blue) is superimposed on the 3D-structure of budding yeast E2 domain (PDB:1JBB, pink). The catalytic Cys residue (red) in the 3D structure of the E2 domain (PDB:1JBB) and its equivalent amino acid residues (blue) in the model structure at the position are highlighted. (i) HeimAB125_07740 (27th-135th amino acid residues) ;(ii) HeimAB125_09840 (1st-107th amino acid residues); (iii) HeimAB125_14070 (1st-130th amino acid residues); (iv) HeimAB125_11700 (25th-122nd amino acid residues). Schematic domain architecture of Asgard E2L proteins is also shown. All structural models were generated using AlphaFold2. **B. Multiple sequence alignment of E2L proteins in Asgard archaea.** Multiple alignment was performed using MUSCLE software.

Supplementary Figure 8. Domain organisation of selected Asgard ESCRT-I proteins. The domains found in ESCRT-I-like proteins of Heimdallarchaeota AB125, Odinarchaeota LCB_4 and Lokiarchaeota GC14_75 are displayed as schematics which highlight the equivalent regions in the previously characterised *S. cerevisiae* ESCRT-I subunits (Ref 1: Kostelansky *et al.* 2007). As a canonical UEV-Vps23 protein coded by Lokiarchaeota GC14_75 has not been identified to date, the correspondent polypeptide of Lokiarchaeota EU551 is represented. The following domains are shown: UEV, Ubiquitin E2 variant domain; 23-Stalk, Vps23 stalk region; 23-SB, Vps23 steadiness box; 28-SB, Vps28 steadiness box; 28-Nt, Vps28 N-terminal extra helices; 28-Ct, Vps28 C-terminal four-helix bundle; RING, RING finger; CC, Coiled coil region; PH, Pleckstrin homology, UBC, Ubiquitin-conjugating enzyme like; PRR, Vps23 proline-rich region; 37-Stalk, Vps37 stalk region; 37-SB, Vps37 steadiness box-like. The total number of residues for each protein is indicated. The following homologous domains are represented with similar colours: 23-Stalk and 37-Stalk; 23-SB, 28-SB and 37-SB; UEV and UBC.

Supplementary Figure 9. Size-exclusion chromatography analyses demonstrating the physical interaction between the Odinarchaeota ubiquitin homologue and the Odinarchaeota Vps23 (TSG101) ESCRT-I subunit. From Top to Bottom: Odinarchaeota ubiquitin protein only (top); Vps23 (TSG101) protein only (middle); Vps23 (TSG101) pre-incubated with ubiquitin, demonstrating stable complex formation (bottom). All proteins were separated on a Superdex S200 HR 10/300 size exclusion chromatography

column. The relative elution volumes of the size standards β-amylase (200 kDa), alcohol dehydrogenase (150 kDa), bovine serum albumin (BSA) (66 kDa) and carbonic anhydrase (29 kDa) and cytochrome-c (12.4 kDa) are also indicated (in grey). Eluted fractions were resolved by SDS-PAGE and visualised by Coomassie stain. Chromatography UV traces (at 280 nm) for the respective elution profiles are displayed to the left of each panel. Three experimental repeats performed with representative experiment displayed.

Supplementary Figure 10. SEC-MALS analyses of the Odinarchaeota ESCRT-I and -II subcomplex proteins. A. Analysis of the Vps23(TSG101) ESCRT-I protein that has a fitted molecular weight of 60.37%(±0.91%), consistent with a stable dimer. **B.** Analysis of the Vps28 ESCRT-I protein that has a fitted molecular weight of 32.48% (±2.49%), consistent with a monomer. Note that a minor larger peak consistent with an unstable and transient dimer is also detectable. **C.** Analysis of the Vps23(TSG101)-Vps28 ESCRT-I complex that has a fitted molecular weight of 88.27% (±0.74%), consistent with a trimeric arrangement based on the Vps23(TSG101) dimer in complex with a Vps28 monomer. **D.** Analysis of the Vps22(EAP30) ESCRT-II protein that has a fitted molecular weight of 28.29% (±3.51%), consistent with a monomer. **E.** Analysis of the Vps25 full-length ESCRT-II protein that has a fitted molecular weight of 26.59% (±2.80%), consistent with a monomer. **F.** Analysis of the N-terminally truncated Vps25 ESCRT-II protein that has a fitted molecular weight of 21.59%(±1.26%), consistent with a monomer. In all panels the differential refractive index (dRI) is plotted in conjunction with molecular weight (M_w). The experimental configuration was calibrated with a BSA standard as detailed in the methods.

Supplementary Figure 11. Chemical crosslinking coupled with MS analysis showed homodimerization of Heimdallarchaeotal Vps22. A. Inter-molecular dimeric peptide identified for HeVps22 using EDC chemical cross-linker. **B.** Intermolecular dimeric peptide was identified for HeVps22 using BS3 chemical cross-linker. Green colour indicates precursor ions. The red and blue colours indicate the b and y fragment ions, respectively. Note that in A the α and β peptides have the same sequence, ruling out the possibility of “intramolecular crosslinks” and suggesting an intermolecular dimeric (or multimeric) configuration. Similarly, in B peptide (KQLKK)FEK is crosslinked to KQLK(KFEK) ruling out the possibility of an intramolecular crosslink and suggesting an intermolecular dimeric (or multimeric) configuration.

Supplementary Figure 12. Modelling of Heimdallarchaeotal Vps25 structure shows conservation with its human counterpart. Heimdall Vps25 structure was generated using AlphaFold2 (Mirdita *et al.,* 2021 [Ref: 2]; Jumper *et al.,* 2021[Ref: 3]). The N and C-terminal structures of human Vps25 (3CUQ and 3HTU) were superimposed with UCSF Chimera.

Supplementary Figure 13. Structure prediction and physical biochemistry of Odinarchaeota Vps25 ESCRT-II protein. Tandem winged-helix (WH) domains in the Odinarchaeota Vps25 ESCRT-II components as demonstrated by structural modelling. (A) (left) Rank1 AlphaFold2 model of the Odinarchaeota Vps25 protein (Mirdita *et al.,* 2021[Ref: 2]; Jumper *et al.,* 2021[Ref: 3]). (middle) Odinarchaeota Vps25ΔN crystal structure solved in this study (PDB: 7PB9), (right) structural superposition of the Odinarchaeota Vps25ΔN crystal structure with the Odinarchaeota AF2 model. (B) (left) AlphaFold2 model of the Odinarchaeota Vps25 protein (Mirdita *et al.,* 2021 [Ref: 2]; Jumper *et al.,* 2021 [Ref: 3]). (middle) *S. cerevisiae* Vps25 crystal structure (PDB: 1XB4), (right) structural superposition of the *S. cerevisiae* Vps25 crystal structure with the Odinarchaeota AF2 model. Also see Figure 4 for alignment of the Odinarchaeota Vps25ΔN crystal structure with the eukaryotic Vps25 homologues from *H. sapiens* (PDB: 2ZME). The alpha-helices in the N-terminal extension unique to the Odinarchaeota Vps25 protein are highlighted in the blue circles. (C) (left) Predicted alignment error of the Rank1 Vps25 AF2 model (right) Predicted lDDT per position (per-residue confidence metric) of the top five Vps25 AF2 models (ranked 1 to 5). (D) Circular dichroism (CD) experimental spectra of the Odinarchaeota Vps25 and N terminally truncated Vps25ΔN ESCRT-II subcomplex proteins. The CD data reveals that the estimated secondary structural elements, based on BeStSel fitting, agree with the predicted models. In each panel, the average spectra are shown for six replicate spectra (triplicate measurements for two identical samples) over a 190-250 nm wavelength range, error bars show the standard deviation of the mean [N = 6]. Summary of the percentage of secondary structural elements

(% alpha helix, % beta-strand, % other) for the Odinarchaeota Vps25 and Vps25ΔN proteins estimated from the CD spectra based on data fitting in BeStSel (All fittings had an NRMSD value < 0.1) [Ref 4: Micsonai *et al*, 2018], or from the predicted structural models (and also the Vps25ΔN crystal structure) using the STRIDE Web-server [Ref 5: Frishman and Argos, 1995]. (E) Thermomelt CD spectra of Odinarchaeota Vps25 and N terminally truncated Vps25ΔN proteins taken in 10°C increments over a 20-90°C temperature range.

Supplementary Figure 14 Figure. Structure prediction and physical biochemistry of Odinarchaeota Vps22 (EAP30 domain) ESCRT-II protein. Tandem winged-helix (WH) domains in the Odinarchaeota Vps22 ESCRT-II components as demonstrated by structural modelling. (A) (left) AlphaFold2 model of the Odinarchaeota Vps22 protein (Mirdita *et al.,* 2021 [Ref: 2]; Jumper *et al.,* 2021 [Ref: 3]). (middle) *H. sapiens* Vps22 crystal structure (PDB: 3CUQ), (right) structural superposition of the *H. sapiens* Vps22 crystal structure with the Odinarchaeota AF2 model. (B) (left) Predicted alignment error of the Rank1 Vps22 (EAP30) AF2 model (right) Predicted lDDT per position (per-residue confidence metric) of the top five Vps22 (EAP30) AF2 models (ranked 1 to 5). (C) Circular dichroism (CD) experimental spectra of the Odinarchaeota Vps22 ESCRT-II subcomplex protein. The CD data reveals that the estimated secondary structural elements, based on BeStSel fitting, agree with the predicted models. The average spectra are shown for six replicate spectra (triplicate measurements for two identical samples) over a 190-250 nm wavelength range, error bars show the standard deviation of the mean [N = 6]. Summary of the percentage of secondary structural elements (% alpha helix, % beta-strand, % other) for the Odinarchaeota Vps22 estimated from the CD spectra based on data fitting in BeStSel (all fittings had an NRMSD value < 0.1) [Ref 4: Micsonai *et a*l, 2018], or from the predicted structural models (and also the Vps25ΔN crystal structure) using the STRIDE Web-server [Ref 5: Frishman and Argos, 1995]. (D) Thermomelt CD spectra of Odinarchaeota Vps22 protein taken in 10°C increments over a 20-90°C temperature range.

Supplementary Figure 15. Y2H assay-based identification of protein-protein interactions amongst fission yeast Ub-ESCRT pathway. A. The reliability of the Y2H assay used in this study was assessed using a group of known eukaryotic Ub-ESCRT pathway genes from the fission yeast *Schizosaccharomyces pombe*. Positive pairs are marked within boxes with broken lines. Bait for Vps28 (*) showed autoactivation but showed stronger β-galactocidase activity when co-expressed with Vps25 (prey). **B.** Schematic representation of interactions detected in the Y2H assay amongst fission yeast Ub-ESCRT proteins.

Supplementary Figure 16. Y2H assay-based identification of Ub-ESCRT protein-protein interactions from 4 different members of the Asgard superphylum. In this figure, interactions detected in both directions (P1-bait: P2-prey and P2-bait: P1-prey) are shown with a green filled circle and those that were observed only in one direction (P1-bait: P2-prey or P2-bait: P1-prey) are shown with a yellow filled circle.

Supplementary References

1. Kostelansky, M. S. *et al.* Molecular architecture and functional model of the complete yeast ESCRT-I heterotetramer. *Cell* **129**, 485-498, doi:10.1016/j.cell.2007.03.016 (2007).
2. Mirdita, M. *et al.* ColabFold - Making protein folding accessible to all. *bioRxiv*, 2021.2008.2015.456425, doi:10.1101/2021.08.15.456425 (2022)
3. Jumper, J. *et al.* Highly accurate protein structure prediction with AlphaFold. *Nature* **596**, 583-589, doi:10.1038/s41586-021-03819-2 (2021).
4. Micsonai, A. *et al.* BeStSel: a web server for accurate protein secondary structure prediction and fold recognition from the circular dichroism spectra. *Nucleic Acids Res* **46**, W315-W322, doi:10.1093/nar/gky497 (2018).
5. Frishman, D. & Argos, P. Knowledge-based protein secondary structure assignment. *Proteins* **23**, 566-579, doi:10.1002/prot.340230412 (1995).

**Data collection**	
Beamline	I03, DLS
Wavelength (Å)	0.9763
Resolution range (Å)§	50.60 – 1.80 (1.85 – 1.80)
Space group	$P2_12_12$
Unit cell parameters (Å, °)	*a* = 101.2, *b* = 31.5, *c* = 59.5, === 90
No. of unique reflections	18,405
Multiplicity§	12.7 (13.2)
Completeness (%)§	100 (100)
Mean I/sigI§	10.3 (1.1)
R_{meas}§§	0.12 (2.56)
R_{pim}	0.05 (0.96)
$CC_{1/2}$§	1 (0.7)
**Refinement**	
Resolution (Å)	50.6 – 1.8
R_{work}††	0.20
R_{free}†	0.26
R.m.s.d., bonds lengths (Å)	0.023
R.m.s.d., angles (°)	2.00
Mean *B* factor (Å²)	36.95
Wilson *B* factor (Å²)	29.8
Ramachandran favoured/outliers	170 / 1 (99.42 %)
Rotamer outliers	0
Molprobity score	1.47

§Parentheses indicate the high resolution shell
§§ $R_{meas} = \{\sum_{hkl}\sqrt{n/(n-1)}[\sum^{n}_{j=1}|I_{hkl,j} - \langle I_{hkl}\rangle|]\}/\sum_{hkl}\sum_j I_{hkl,j}$
††$Rwork = \sum||Fobs| - |Fcalc||/|Fobs| \times 100$
† Rfree, based on 5% of the total reflections

Supplementary Table 1. Data collection and refinement statistics for the Odinarchaeota Vps25ΔN (58-228 aa) crystal structure (PDB: 7PB9).

Purpose	Protein/Target	Gene name	Amino acid region	AA substitution	Entry	Name	Sequence
Protein purification	Heimdall Ubiquitin	HeimAB125_14240	1:77 aa	None	**TH15-65**	pET28a:HeUb_f	GCCTGGTGCCGCGCGGCAGCATGCGTATTACAGTTGTTACAGCTATAGGC
					**TH15-66**	HeUb:pET28a_r	TGTCCACCAGTCATGCTAGCTTAgccgccTTTGGTGCGAGCTATTAAGTAACATTTGTCACC
Protein purification	Heimdall Ubiquitin	HeimAB125_14240	1:77 aa	V45D	**TH15-65**	pET28a:HeUb_f	GCCTGGTGCCGCGCGGCAGCATGCGTATTACAGTTGTTACAGCTATAGGC
					**TH15-66**	HeUb:pET28a_r	TGTCCACCAGTCATGCTAGCTTAgccgccTTTGGTGCGAGCTATTAAGTAACATTTGTCACC
Protein purification	Heimdall UEV-Vps28	HeimAB125_14070	Full length	None	**TH12-53**	SUMO:He14070_f	TGCTGCACCAAACTGGAGGCATGAACAAGATTTCTACCGATGATTTCTTGCTTGC
					**TH12-54**	He14070:pET28a_r	CGAGTGCGGCCGCAAGCTTGttaAAGCTCTTTAAGAACACGACGAAAATCATTAAGC
Protein purification	Heimdall UEV-Vps28	HeimAB125_14070	1:130 aa	None	**TH12-53**	SUMO:He14070_f	TGCTGCACCAAACTGGAGGCATGAACAAGATTTCTACCGATGATTTCTTGCTTGC
					**TH16-13**	He14070delC_130aa_R	CGAGTGCGGCCGCAAGCTTGttaAGTGTAACGTTGTTCCTCTTTAGGC
Protein purification	Heimdall Vps22	HeimAB125_14050	Full length	None	**TH12-51**	SUMO:He14050_f	TGCTGCACCAAACTGGAGGCATGGGTTTACGTGATATTGAGAAGAAGATCAAGG
					**TH12-52**	He14050:pET28a_r	CGAGTGCGGCCGCAAGCTTGttaAAGGCCCATTAAACCAGGAAACCAAAACTTAAC
Y2H (bait/prey)	S.pombe Ubiquitin	ubi4	1:76 aa	None	**TH15-38**	pMM_ubi4rp1_f	GTTCTGGCCCCGGTCAGGGAATGCAGATTTTCGTCAAGACATTAACTGG
					**TH15-39**	pMM_ubi4rp1_r	TATCGAATTCCTGCAGCCCGttaACCGCCACGAAGACGAAGAACAAGG
Y2H (bait/prey)	S.pombe Vps23	sst6 (vps23)	Full length	None	**TH15-36**	pMM_sst6_f	GTTCTGGCCCCGGTCAGGGAATGTCAGATCATGCAATCAATGAACACC
					**TH15-37**	pMM_sst6_r	TATCGAATTCCTGCAGCCCGTTAAGAGTATTGAAGCATTTCGTCCCG
Y2H (bait/prey)	S.pombe Vps28	vps28	Full length	None	**TH15-48**	pMM_vps28_f	GTTCTGGCCCCGGTCAGGGAATGACTGAATACTACGATCTGAACCTTTTAGAAAAGG
					**TH15-49**	pMM_vps28_r	TATCGAATTCCTGCAGCCCGTCATAACAAGCTATAGCACTCTGCGTAGGC
Y2H (bait/prey)	S.pombe Vps36	vps36	Full length	None	**TH15-52**	pMM_vps36_f	GTTCTGGCCCCGGTCAGGGAATGGCCTTTTATTTAGAAACAACCCCTTCTAATCTACC
					**TH15-53**	pMM_vps36_r	TATCGAATTCCTGCAGCCCGCTATGATTCATCAAACCATTTAGTCAATTCTTCTGATAATTCGTCAAATTCG
Y2H (bait/prey)	S.pombe Vps25	vps25	Full length	None	**TH15-46**	pMM_vps25_f	GTTCTGGCCCCGGTCAGGGAATGCGTGTTCCATCCATTTATAACTTTCC
					**TH15-47**	pMM_vps25_r	TATCGAATTCCTGCAGCCCGTTAAGCTTTCAGTACCTTGAACCCGCTATATTTTCC
Y2H (bait/prey)	S.pombe Vps22	dot2 (vps22)	Full length	None	**TH15-34**	pMM_dot2_f	GTTCTGGCCCCGGTCAGGGAATGCGTAAAAGAATTGGAATTGGAGCTTTGAACG
					**TH15-35**	pMM_dot2_r	TATCGAATTCCTGCAGCCCGTTAGAAATTTTCGTATAAAAACCTTTGCGCCTGG
Y2H (bait/prey)	S.pombe ESCRT-III	cmp7	Full length	None	**TH15-28**	pMM_cmp7_f	GTTCTGGCCCCGGTCAGGGAATGGGAAAAAAGGAGAAAGTCAATGCTTTGG
					**TH15-29**	pMM_cmp7_r	TATCGAATTCCTGCAGCCCGTTATTGCTCAACCAGCTCAGCTTGAATCTTCTCC
Y2H (bait/prey)	S.pombe ESCRT-III	did2	Full length	None	**TH15-30**	pMM_did2_f	GTTCTGGCCCCGGTCAGGGAATGAGCAATTTAGAGGCATCTCTGTTTCAATTAAAGTTTGC
					**TH15-31**	pMM_did2_r	TATCGAATTCCTGCAGCCCGTCAAGACCTTAAAGCACGTAAACGTTCTTG
Y2H (bait/prey)	S.pombe ESCRT-III	did4	Full length	None	**TH15-32**	pMM_did4_f	GTTCTGGCCCCGGTCAGGGAATGGGCTTAACTTCTTGGCTATTTGG
					**TH15-33**	pMM_did4_r	TATCGAATTCCTGCAGCCCGTTAACGCTTTGCCAGTTCATCCAATCTGG
Y2H (bait/prey)	S.pombe ESCRT-III	vps20	Full length	None	**TH15-42**	pMM_vps20_f	GTTCTGGCCCCGGTCAGGGAATGGGGGTTAACAGTAGTAAAATTAATGATAAGG
					**TH15-43**	pMM_vps20_r	TATCGAATTCCTGCAGCCCGTTATCCCAGGACTGGATCTTTTCGAAGTTC
Y2H (bait/prey)	S.pombe ESCRT-III	vps24	Full length	None	**TH15-44**	pMM_vps24_f	GTTCTGGCCCCGGTCAGGGAATGCAAACTGTAAGATCTTATTTCTTTGGTCCG
					**TH15-45**	pMM_vps24_r	TATCGAATTCCTGCAGCCCGTTAGGATTTCAAAGCATCTAGCTTATCACGAATGTCC
Y2H (bait/prey)	S.pombe ESCRT-III	vps32	Full length	None	**TH15-50**	pMM_vps32_f	GTTCTGGCCCCGGTCAGGGAATGTCTGGATTTTTAAGATGGTTTGGGGGC
					**TH15-51**	pMM_vps32_r	TATCGAATTCCTGCAGCCCGTTAAAGAGAGAATTCAGCCTGTAGTTTTCGAAGTTCTTCTTCTTC
Y2H (bait/prey)	S.pombe Vps4	vps4	Full length	None	**TH15-40**	pMM_vps4_f	GTTCTGGCCCCGGTCAGGGAATGTCCAATCCAGATTGTTTAAGTAAAGCAATTTC
					**TH15-41**	pMM_vps4_r	TATCGAATTCCTGCAGCCCGCTATCCCTCTGCTCCAAAGTCCTTTGTAAATTGTG
Y2H (bait/prey), universal	Asgard archaea ESCRT	HeimAB125/Lokiarch/ThorAB25/OdinLCB4	Full length	None	**Primer585**	Y2H-As-ESCRT-Fw	GGGATCCCCCGGGCTGCAGGAttagaaggttctggtcaaggtcctg
					**Primer586**	Y2H-As-ESCRT-Rv	TGACATAACTAATTACATGACGGTAAAAGATgcggccgcTTAca
Protein purification	Odinarchaeota Ubiquitin	OdinLCB4_14240 Ubiquitin	Full length	None		OdUBQforNdeI	GCGCATATGAAAATAGAAATTGTTTCAGCTATAGGTG
						OdinUbqGGSTOPrevXhoI	GCGCTCGAGCTACCCCCCTTCAGTTCTGGTAATTAAATATACCCTATCC
Protein purification	Odinarchaeota Vps23	OdinLCB4_14270 Vps23 (TSG101) ESCRT-I	Full length	None		OdV23TSGE2forNdeI	GCGCATATGGACACCTCTATCATATATCAGGAAGCGGAATTAATGTATAAAATGG
						OdV23TSGE2revXhoI	GCGCTCGAGATTTTTAGATTCTGCTTTACGAGTATCGGAAAGGTTTTTCCCAACTAAAAACAAC
						OdV23TSG101revstop	GTGCTCGAGTCAATTTTTAGATTCTGCTTTACGAGTATC
Protein purification	Odinarchaeota Vps28	OdinLCB4_14280 Vps28 ESCRT-I	Full length	None		OdESCIV28forNdeIshotgun	GCGCATATGCAAGAAAAAAATTGTGTAAAAGAATCTGAGGAACG
						OdESCIV28revXhoI	GCGCTCGAGCATACATGCTTTTTTAATCATGCATTCAAATTCATTTCTCCAGGTTTGAATAG
						Vps28intNdeImut	CATTAGAGTCACCTGGGCGAATAGCTGTTAAAACTTATGAGTTAGCTTCAAGTTTTATC
						Vps28Stopxhorev	GTGCTCGAGTCACATACATGCTTTTTTAATCATGCATTCAAATTC
Protein purification	Odinarchaeota Vps22	OdinLCB4_14290 Vps22 (EAP30) ESCRT-II	Full length	None		OdESCIIEAP30forNdeI	GCGCATATGGGTATCAGCGAGATAGAAAACCGGTTAAAAACTAAAAGAGC
						OdESCIIEAP30revXhoI	GCGCTCGAGTTTGCTTAGAAAAGCAGGGAAATAATATTTCTTACCATGAGCGTAAC
						OdESCIIEAP30stoprevXhoI	GCGCTCGAGTCATTTGCTTAGAAAAGCAGGGAAATAATATTTCTTACCATGAGCGTAAC
Protein purification	Odinarchaeota Vps25	OdinLCB4_14300 Vps25 ESCRT-II	Full length	None		OdESCIIV25forNdeI	GCGCATATGGAGCTCACCAAGCGTAGAGATAAAAACACCC
						OdESCIIV25revXhoI	GCGCTCGAGGAATAATATTCTTATATGCATGTTTTTTTTATTAATCCATTCCGC
			58-228 aa			OdNTrunc_ESCIIV25forNdeI	GCGCATATGTTAACTGTAGGATTTGAATGGGCCGC

Supplementary Table 2. Oligonucleotides used in the generation of expression constructs used in this study.

OdinLCB4 Y2H plasmids			***Lokiarch Y2H plasmids***			***HeimAB125 Y2H plasmids***			***S.pombe Y2H plasmids***		
Collection #	Vector	Gene	Collection #	Vector	Gene	Collection #	Vector	Gene	Collection #	Vector	Gene
pSPW378	pMM5	**Ubiquitin**	pSPW376	pMM5	**Ubiquitin**	pSPW374	pMM5	**Ubiquitin**	pSPW738	pMM5	**SpUbi4**
pSPW366	pMM5	**Vps23**	pSPW364	pMM5	**Vps23**	pSPW362	pMM5	**Vps23**	pSPW739	pMM5	**SpSst6**
pSPW334	pMM5	**Vps28**	pSPW332	pMM5	**Vps28**	pSPW330	pMM5	**Vps28**	pSPW740	pMM5	**SpVps28**
pSPW344	pMM5	**Vps22**	pSPW336	pMM5	**SB**	pSPW340	pMM5	**Vps22**	pSPW741	pMM5	**SpVps36**
pSPW380	pMM5	**Vps25**	pSPW342	pMM5	**Vps22**	pSPW346	pMM5	**Vps25**	pSPW742	pMM5	**SpVps22(Dot2)**
pSPW354	pMM5	**ESCRT-III (A)**	pSPW348	pMM5	**Vps25**	pSPW350	pMM5	**ESCRT-III (A)**	pSPW743	pMM5	**SpVps25**
pSPW360	pMM5	**ESCRT-III (B)**	pSPW352	pMM5	**ESCRT-III (B)**	pSPW356	pMM5	**ESCRT-III (B)**	pSPW744	pMM5	**SpDid2**
pSPW368	pMM5	**Vps4**	pSPW358	pMM5	**ESCRT-III (A)**	pSPW478	pMM5	**ESCRT-III (B) 52C**	pSPW745	pMM5	**SpVps20**
pSPW430	pMM6	**Ubiquitin**	pSPW372	pMM5	**Vps4**	pSPW370	pMM5	**Vps4**	pSPW746	pMM5	**SpVps32**
pSPW418	pMM6	**Vps23**	pSPW428	pMM6	**Ubiquitin**	pSPW426	pMM6	**Ubiquitin**	pSPW747	pMM5	**SpVps1(Did4)**
pSPW386	pMM6	**Vps28**	pSPW416	pMM6	**Vps23**	pSPW414	pMM6	**Vps23**	pSPW748	pMM5	**SpVps24**
pSPW396	pMM6	**Vps22**	pSPW384	pMM6	**Vps28**	pSPW382	pMM6	**Vps28**	pSPW749	pMM5	**SpVps4**
pSPW432	pMM6	**Vps25**	pSPW388	pMM6	**SB**	pSPW392	pMM6	**Vps22**	pSPW750	pMM5	**SpCmp7**
pSPW406	pMM6	**ESCRT-III (A)**	pSPW394	pMM6	**Vps22**	pSPW398	pMM6	**Vps25**	pSPW751	pMM6	**SpUbi4**
pSPW412	pMM6	**ESCRT-III (B)**	pSPW400	pMM6	**Vps25**	pSPW402	pMM6	**ESCRT-III (A)**	pSPW752	pMM6	**SpSst6**
pSPW420	pMM6	**Vps4**	pSPW404	pMM6	**ESCRT-III (B)**	pSPW408	pMM6	**ESCRT-III (B)**	pSPW753	pMM6	**SpVps28**
			pSPW410	pMM6	**ESCRT-III (A)**	pSPW478	pMM6	**ESCRT-III (B) 52C**	pSPW754	pMM6	**SpVps36**
			pSPW424	pMM6	**Vps4**	pSPW422	pMM6	**Vps4**	pSPW755	pMM6	**SpVps22(Dot2)**
									pSPW756	pMM6	**SpVps25**
									pSPW757	pMM6	**SpDid2**
									pSPW758	pMM6	**SpVps20**
									pSPW759	pMM6	**SpVps32**
									pSPW760	pMM6	**SpVps1(Did4)**
									pSPW761	pMM6	**SpVps24**
									pSPW762	pMM6	**SpVps4**
									pSPW763	pMM6	**SpCmp7**

Supplementary Table 3. Details of all Yeast-2-Hybrid (Y2H) constructs generated and used in this study.
