# Supplementary material for: Asgard archaea shed light on the evolutionary origins of the eukaryotic ubiquitin-ESCRT machinery
Source: Nat Commun. 2022 Jun 13;13:3398. doi: 10.1038/s41467-022-30656-2 (PMC9192718; doi:10.1038/s41467-022-30656-2)
Supplement: Supplementary file 2 — Reporting Summary [file 41467_2022_30656_MOESM2_ESM.pdf]

nature portfolio

Corresponding author(s): Mohan Balasubramanian, Nicholas P Robinson and Buzz Baum

Last updated by author(s): May 5, 2022

Reporting Summary

Nature Portfolio wishes to improve the reproducibility of the work that we publish. This form provides structure for consistency and transparency in reporting. For further information on Nature Portfolio policies, see our Editorial Policies and the Editorial Policy Checklist.

Statistics

For all statistical analyses, confirm that the following items are present in the figure legend, table legend, main text, or Methods section.

n/a	Confirmed	
☐	☒	The exact sample size (*n*) for each experimental group/condition, given as a discrete number and unit of measurement
☒	☐	A statement on whether measurements were taken from distinct samples or whether the same sample was measured repeatedly
☐	☒	The statistical test(s) used AND whether they are one- or two-sided<br>*Only common tests should be described solely by name; describe more complex techniques in the Methods section.*
☒	☐	A description of all covariates tested
☒	☐	A description of any assumptions or corrections, such as tests of normality and adjustment for multiple comparisons
☒	☐	A full description of the statistical parameters including central tendency (e.g. means) or other basic estimates (e.g. regression coefficient) AND variation (e.g. standard deviation) or associated estimates of uncertainty (e.g. confidence intervals)
☒	☐	For null hypothesis testing, the test statistic (e.g. *F*, *t*, *r*) with confidence intervals, effect sizes, degrees of freedom and *P* value noted<br>*Give P values as exact values whenever suitable.*
☒	☐	For Bayesian analysis, information on the choice of priors and Markov chain Monte Carlo settings
☒	☐	For hierarchical and complex designs, identification of the appropriate level for tests and full reporting of outcomes
☒	☐	Estimates of effect sizes (e.g. Cohen's *d*, Pearson's *r*), indicating how they were calculated

Our web collection on statistics for biologists contains articles on many of the points above.

Software and code

Policy information about availability of computer code

Data collection

X-ray diffraction data collection
Native diffraction data were collected at Diamond Light Source (Harwell, UK) at beamline I03. Data were collected over 360° with 0.1° oscillation (Supplementary Table 1), integrated with DIALS131 and scaled/merged with Aimless132 from the CCP4 suite133.

Data analysis

Genomic survey of protein homologues: Prodigal v2.6.3, Mafft-linsi v7.450, trimAl v1.4.rev22, IQ-Tree v2.0-rc1
Co-location of these genes was investigated through custom perl scripts and visualized using R110 and the packages ggplot2111, cowplot112, and genoPlotR113

AlphaFold2 on https://colab.research.google.com/github/sokrypton/ColabFold/blob/main/AlphaFold2.ipynb

The Heimdall-, Loki-, Odin-, and Thorarchaeota amino acid sequences were obtained from Uniprot (https://www.uniprot.org/) and the Uniprot entry IDs are listed in Supplementary Data 1.
Phylogenetic reconstruction. Amino acid sequences were obtained from Uniprot.
sequences were aligned with Mafft-linsi v7.450, and the resulting multiple-sequence alignments were used as query for a Psiblast (v2.10.0+) and trimmed using trimAl v1.4.rev22. Obtained alignments were used for a phylogenetic reconstruction with IQ-Tree 2.0-rc2107, under the model Q.pfam+C20+G4+F, chosen by ModelFinder122 between combinations of empirical matrices (LG, WAG, JTT, and Q.pfam) with mixture models (C20, C40, and C60) and various rate heterogeneity (none, G4 and R4) and frequency (none, and F) and using 1000 ultrafast bootstrap pseudoreplicates. The resulting phylogeny was used as guide to reconstruct another tree under the PMSF approximation of the chosen model and using 100 non-parametric bootstrap pseudoreplicates. The resulting bootstrap trees were used both using the standard Felsenstein Bootstrap Proportion and the more recent Transfer Bootstrap Expectation123 interpretations.

SEC-MALS analyses in Wyatt's ASTRA software

CD spectra were interpolated using Origin Pro 2018b and fitted to the BeStSel algorithm and the STRIDE web server128 to determine the secondary structural elements.

X-ray diffraction Data were integrated with DIALS131 and scaled/merged with Aimless132 from the CCP4 suite133. BALBES was used to determine initial phases by Molecular Replacement against the entire PDB134. Manual building was done in COOT135 and refinement with REFMAC5. MOLPROBITY was used for model validation136.

Mass spectrometry. The ProteoWizard MSConvert toolkit42 was used to convert the raw data files into .mgf format. Scaffold Proteome Software was used for sequence visualization and coverage. Cross-linked peptides were analysed using the Stavrox software137

For manuscripts utilizing custom algorithms or software that are central to the research but not yet described in published literature, software must be made available to editors and reviewers. We strongly encourage code deposition in a community repository (e.g. GitHub). See the Nature Portfolio guidelines for submitting code & software for further information.

Data

Policy information about availability of data

All manuscripts must include a data availability statement. This statement should provide the following information, where applicable:

- Accession codes, unique identifiers, or web links for publicly available datasets
- A description of any restrictions on data availability
- For clinical datasets or third party data, please ensure that the statement adheres to our policy

All amino acid sequences referred to in this study were obtained from Uniprot (https://www.uniprot.org/) and the Uniprot entry IDs are listed in Supplementary Data 1.
7PB9 is the PDB accession code for the OdinarchaeotaN_Vps25 structure solved in this study deposited in the Protein Data Bank.
Other previously published PDB accessions referred to in this study: 1XB4, 3CUQ, 3HTU, 6VME, 2J9U, 1JBB, 1UZX

Field-specific reporting

Please select the one below that is the best fit for your research. If you are not sure, read the appropriate sections before making your selection.

☒ Life sciences ☐ Behavioural & social sciences ☐ Ecological, evolutionary & environmental sciences

For a reference copy of the document with all sections, see nature.com/documents/nr-reporting-summary-flat.pdf

Life sciences study design

All studies must disclose on these points even when the disclosure is negative.

Sample size	Following standard rules for sample size: N=3 for the biochemical analyses presented.
Data exclusions	No data were excluded from these analyses.
Replication	Triplicate measurements for the biochemical analyses presented.
Randomization	There was no data that required randomization
Blinding	There was no data that required blinding

Reporting for specific materials, systems and methods

We require information from authors about some types of materials, experimental systems and methods used in many studies. Here, indicate whether each material, system or method listed is relevant to your study. If you are not sure if a list item applies to your research, read the appropriate section before selecting a response.

Materials & experimental systems

n/a	Involved in the study
☒	☐ Antibodies
☒	☐ Eukaryotic cell lines
☒	☐ Palaeontology and archaeology
☒	☐ Animals and other organisms
☒	☐ Human research participants
☒	☐ Clinical data
☒	☐ Dual use research of concern

Methods

n/a	Involved in the study
☒	☐ ChIP-seq
☒	☐ Flow cytometry
☒	☐ MRI-based neuroimaging
