# Supplementary material for: Asgard archaea shed light on the evolutionary origins of the eukaryotic ubiquitin-ESCRT machinery
Source: Nat Commun. 2022 Jun 13;13:3398. doi: 10.1038/s41467-022-30656-2 (PMC9192718; doi:10.1038/s41467-022-30656-2)
Supplement: Supplementary file 4 — Description of Additional Supplementary Files [file 41467_2022_30656_MOESM4_ESM.pdf]

Title: Supplementary Data 1:
Description: Predicted amino acid sequences of Asgard ESCRT proteins used in this study. Predicted amino acid sequences generated by in silico translation of the coding DNA sequences of the Asgard ESCRT components investigated in this study.

Title: Supplementary Data 2:
Description: Mass spectrometry identifying intra vps22 crosslinks. Shown are all the crosslinked peptides recovered after mass spectrometry. The columns specify the sequence of Peptide 1 and Peptide 2 participating in the crosslink as well as the crosslinked residue in each of the peptides. Also shown in each row is the identity of the crosslinker.
